# Geobiology reveals how human kidney stones dissolve *in vivo*

**DOI:** 10.1038/s41598-018-31890-9

**Published:** 2018-09-13

**Authors:** Mayandi Sivaguru, Jessica J. Saw, James C. Williams, John C. Lieske, Amy E. Krambeck, Michael F. Romero, Nicholas Chia, Andrew L. Schwaderer, Reinaldo E. Alcalde, William J. Bruce, Derek E. Wildman, Glenn A. Fried, Charles J. Werth, Richard J. Reeder, Peter M. Yau, Robert A. Sanford, Bruce W. Fouke

**Affiliations:** 1 Carl R. Woese Institute for Genomic Biology, University of Illinois at Urbana-Champaign, Urbana, IL USA; 2 Carl Zeiss Labs@Location Partner, Carl R. Woese Institute for Genomic Biology University of Illinois at Urbana-Champaign, Urbana, IL USA; 3 Department of Molecular and Integrative Physiology, University of Illinois at Urbana- Champaign, Urbana, IL USA; 4 Mayo Clinic School of Medicine, Mayo Clinic, Rochester, MN USA; 5 Department of Anatomy and Cell Biology, Indiana University School of Medicine, Indianapolis, IN USA; 6 Division of Nephrology and Hypertension, Mayo Clinic, Rochester, MN USA; 7 Department of Laboratory Medicine and Pathology, Mayo Clinic, Rochester, MN USA; 8 Department of Urology, Mayo Clinic, Rochester, MN USA; 9 Department of Urology, Indiana University School of Medicine, Indianapolis, IN USA; 10 Department of Physiology & Biomedical Engineering, Mayo Clinic, Rochester, MN USA; 11 Department of Individualized Medicine, Mayo Clinic, Rochester, MN USA; 12 Department of Pediatric Nephrology, Indiana University School of Medicine, Indianapolis, IN USA; 13 Civil, Architectural and Environmental Engineering, University of Texas at Austin, Austin, TX USA; 14 Loyola University Chicago, Stritch School of Medicine, Maywood, IL USA; 15 Department of Geosciences, Stony Brook University, Stony Brook, NY 11794 USA; 16 Protein Sciences, Roy J. Carver Biotechnology Center, University of Illinois at Urbana-Champaign, Urbana, IL USA; 17 Department of Geology, University of Illinois at Urbana-Champaign, Urbana, IL USA; 18 Department of Microbiology, University of Illinois at Urbana-Champaign, Urbana, IL USA; 19 Roy J. Carver Biotechnology Center, University of Illinois at Urbana-Champaign, Urbana, IL USA

## Abstract

More than 10% of the global human population is now afflicted with kidney stones, which are commonly associated with other significant health problems including diabetes, hypertension and obesity. Nearly 70% of these stones are primarily composed of calcium oxalate, a mineral previously assumed to be effectively insoluble within the kidney. This has limited currently available treatment options to painful passage and/or invasive surgical procedures. We analyze kidney stone thin sections with a combination of optical techniques, which include bright field, polarization, confocal and super-resolution nanometer-scale auto-fluorescence microscopy. Here we demonstrate using interdisciplinary geology and biology (*geobiology*) approaches that calcium oxalate stones undergo multiple events of dissolution as they crystallize and grow within the kidney. These observations open a fundamentally new paradigm for clinical approaches that include *in vivo* stone dissolution and identify high-frequency layering of organic matter and minerals as a template for biomineralization in natural and engineered settings.

## Introduction

Kidney stones have been formally studied by western medicine since 1802^1,2^. In 1868, Beale^3^ reported that urine contains suspended crystal sediments of calcium oxalate (CaOx, oxalate of lime) that are “difficult” to dissolve. Research then focused on mineralogy, chemistry and protein incorporation as primary controls on stone growth, with little evaluation of urine chemistry^4–6^. These minerals originally form as tetragonal bipyramid crystals of CaOx dihydrate (COD; *Weddellite*; CaC_2_O_4_ • 2H_2_O) that “transform during the partial loss of water” into monoclinic lozenge-shaped crystals of CaOx monohydrate (COM; *Whewellite*; CaC_2_O_4_ • H_2_O)^7–9^. A third common but minor mineral phase in CaOx stones is orthorhombic crystalline uric acid (UA; C_5_H_4_N_4_O_3_)^10^. During the 1960s, stone research transitioned to focus on the role of 24-hour urine solution chemistry and calculated supersaturation as an index of stone formation risk^11^. It is now known that a complex variety of factors other than simple urine chemistry influence stone formation, including protein degradation products, cellular responses, and even the likely presence of a kidney microbiome^12–14^. Moreover, a host of complex human health conditions including dehydration, pregnancy, diet, diabetes, hypertension, obesity, genetics, and climate^12,15^ also affects kidney stone susceptibility. Thus, current clinical strategies to employ hydration, diet and/or drugs to “correct” urine chemistry are at best only partially effective in preventing stone growth.

In the present study, more than 50 CaOx kidney stone fragments, collected from six Mayo Clinic patients during standard percutaneous nephrolithotomy (PCNL) procedures, are analyzed. Integrated geology and biology (*geobiology*) techniques are applied within the chronological and spatial framework of crystal growth and stratigraphic layering patterns (*crystalline architecture*) that comprise each stone fragment. Bulk mineralogical analyses with infrared (IR) spectroscopy confirm that all samples are primarily composed of CaOx (see Methods). We analyze ~20 μm-thick stone fragment sections polished on both sides using a wide range of optical microscopy (250 nm-resolution), which include brightfield, phase contrast, polarization, single- and two-photon spectral confocal, and fluorescence lifetime imaging. In addition, we use Airyscan super-resolution microscopy (~140 nm-resolution) to bring the optical resolution beyond the diffraction limit of light^16–18^. To determine mineralogy and chemistry in thin section, we use scanning electron microscopy and energy-dispersive x-ray spectroscopy. Only stone fragments from patient MP2 contain all events of crystalline architecture collectively identified in all six patient specimens. Therefore, we present MP2 as an exemplar of CaOx kidney stone growth.

Our microscopy analyses indicate that extensive and repeated dissolution occurs throughout the growth history of each stone. This evidence stands in stark contrast to the common perception that kidney stones do not dissolve in the human kidney^3,12,15^. These results suggest multiple novel strategies targeting *in vivo* dissolution may be effective in alleviating the adverse health impacts of this increasingly common disease. At the same time, these insights broaden and advance our ability to accurately interpret the physical, chemical and biological processes that control mineral deposition in a variety of other human diseases, as well as many other natural and engineered environments where biomineralization takes place.

## Results and Discussion

### Historical sequence of events

**Figure 1:**
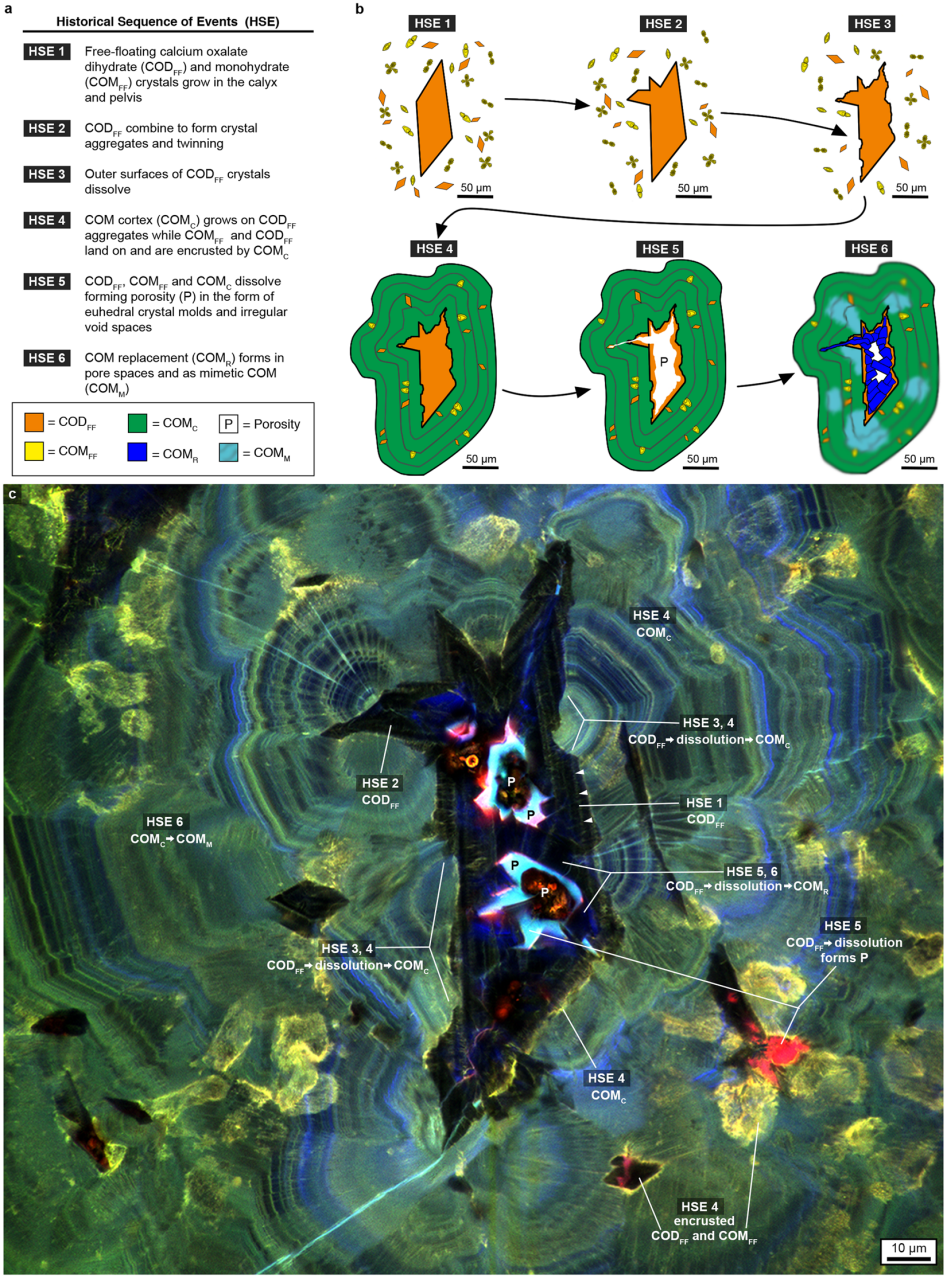
Historical sequence of events (HSE) constructed from super-resolution auto-fluorescence (SRAF) images of the MP2 calcium oxalate (CaOx) kidney stone. (**a**) The HSE. (**b**) Sketch depicting individual HSE events. (**c**) Representative SRAF image composed of merged three pseudo-colored red, green and blue (RGB) channels. Brightness and contrast of the RGB channel intensities are adjusted to highlight the dark crystalline fabrics. Raw images with and without adjustments are presented in Supplementary Fig. 2.

Distinct stratigraphic layering on the scale of 10’s to 100’s of nanometers (*nano-layering*) is revealed by auto-fluorescence (AF, emission of a specific fluorescence light without labels in response to a specific excitation wavelength)^16,17^ generated by changes in organic matter composition (Supplementary Fig. 1). We interpret the crystalline architecture of COD, COM and UA in kidney stones using the Law of Superposition (i.e., older layers at the bottom and younger layers at the top), proposed in 1667 by Nicholas Steno, a Danish physician and pioneering geobiologist^2,19^. Our observations are synthesized into a historical sequence of events (HSE, Fig. 1, a *paragenetic sequence* in geology)^20^.

**Figure 2:**
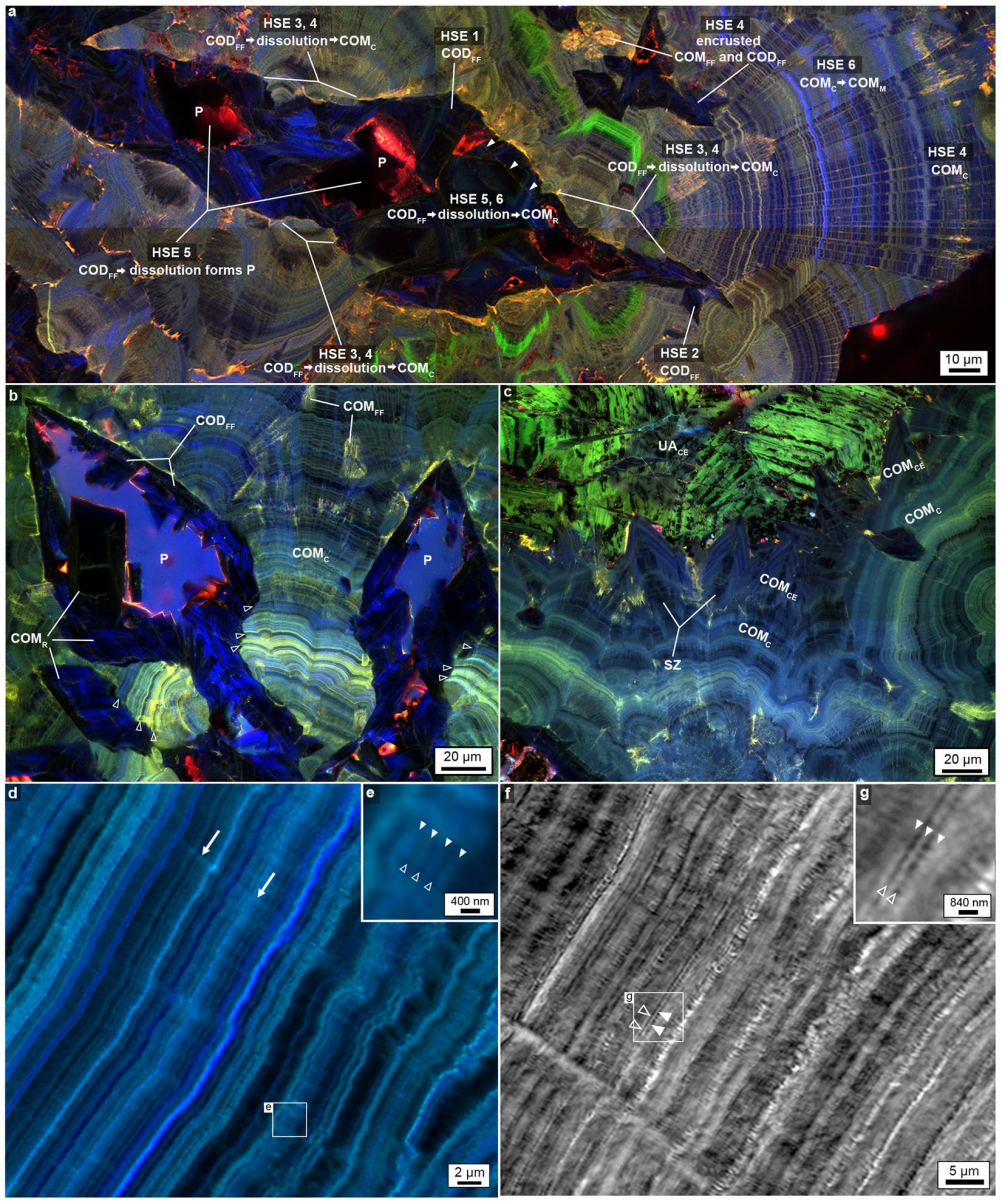
Evidence for *in vivo* dissolution and nano-layering from confocal auto-fluorescence (CAF) and SRAF imaging of the MP2 CaOx kidney stone. Specific areas of the MP2 stone from which these image enlargements are made are shown in Supplementary Fig. 1. (**a**) Tiled CAF image of merged pseudo-colored RGB channels with no image adjustments. (**b**,**c**) SRAF images of merged pseudo-colored RGB channels. The brightness and contrast of each image is individually adjusted to highlight the layered crystalline architecture (raw images with and without adjustments are presented in Supplementary Fig. 22). (**d**) SRAF image of COM_C_ nano-layering from merged two-channel blue and red (pseudo-colored cyan) channels (Z-stack optical sections of all layers are presented in Supplementary Video 2). The SRAF green channel is identical to the red channel (pseudo-colored cyan) and therefore not included. (**e**) Individual ~140 nm-thick dark and light nano-layers (open and closed arrowheads) with enlargement (**e**). Radiating twinned crystals grow with their c-axis oriented perpendicular to each dark or light nano-layer (arrows). (**f**) Black and white circular polarization phase contrast (CPOLPC) image shows COM_C_ dark organic matter-rich and light mineral-rich nano-layering with enlargement (**g**). The original color image of the same area is presented in Supplementary Fig. 18. Images (**d**,**e**) are displayed with best-fit intensity profiles. Images (**f**,**g**) are displayed with best-fit intensity profiles after a gamma correction of 0.4.

The earliest stage of kidney stone growth begins with the precipitation of 5–250 μm-diameter perfectly geometrically formed (*euhedral*) COD and COM with internal concentric zonations consistent with free-floating growth in the renal calyx and/or pelvis (i.e., *crystalline sediment* or *crystalluria*)(COD_FF_ and COM_FF_, HSE 1 in Figs. 1 and 2a)^21,22^. The outermost surfaces of larger COD_FF_ have euhedral extensions reflecting crystal twinning^21^, including the adherence of smaller COD_FF_ to other larger COD_FF_ faces^23^ in the form of aggregates (HSE 2 in Figs. 1, 2a and Supplementary Fig. 2a). These outermost surfaces then dissolve (HSE 3 in Figs. 1 and 2a), cutting 10’s of microns down into internal concentric COD_FF_ layers. Following dissolution, small <5–10 µm-diameter COM_FF_ land on and encrust outer COD_FF_ surfaces (Figs. 1c and 2a). The next generation of COM exhibits a dense nano-layered cortex (COM_C_; HSE 4 in Figs. 1, 2a and Supplementary Fig. 2b)^24,25^ that encrusts both pristine and dissolved COD_FF_ surfaces. In addition, some COM_FF_ land on and are then encrusted by the same COM_C_ (HSE 4 in Figs. 1 and 2a). COM_C_ generally adopts the same crystallographic C-axis orientation as the COD_FF_ and COM_FF_ (Fig. 1c) they encrust (*syntaxial overgrowths*).

The COD_FF_, COM_FF_ and COM_C_ crystal complex then extensively and repeatedly dissolves (HSE 5 in Figs. 1, 2a), which is recorded by four types of fabrics that cross-cut the crystalline architecture (Supplementary Fig. 2a–d): (1) Micron-sized and larger crystals of COD_FF_ partially dissolve from their center, creating irregular void spaces lined with remnants of original COD_FF_ (white arrowheads in Figs. 1c, 2a, Supplementary Fig. 2c). (2) Bulk dissolution completely removes the original COM_C_-encrusted COD_FF_, leaving euhedral mold-shaped void spaces (*moldic porosity*, P in Figs. 1, 2 and Supplementary Fig. 2a–d). (3) Continued bulk dissolution cuts into COM_C,_ creating canyon-like void spaces that cross-cut COM_C_ nano-layering (Fig. 2b and Supplementary Fig. 2d. (4) Both irregular and euhedral moldic porosity within COD_FF_, COM_FF_, and COM_C_ is then partially to completely filled with replacement COM (COM_R_; HSE 6 in Figs. 1, 2a,b and Supplementary Fig. 2b,c,e–g). In addition, Ångstrom-scale dissolution and crystallization (*mimetic replacement*, COM_M_) of COM_C_ takes place, in which the original fine laminations of the cortex are completely to partially preserved (HSE 6 in Figs. 1, 2a and Supplementary Fig. 3).

The final stage of stone growth occurs when three separate stone fragments come into contact, locally dissolve at contact points and interlock to create a larger stone complex (Supplementary Fig. 1b,c and Video 1). At present, we cannot distinguish if the three stone fragments formed entirely by naturally occurring events within the patient’s kidney, or whether they broke apart as a result of previous medical intervention. The margins of each fragment are irregular, intertwined and exhibit large-scale 300–400 µm-scale truncation of COM_C_ nano- layering (Supplementary Fig. 1b,d). These data indicate that dissolution continues as the margins of the stones come into contact. After the fragments merge into a stone complex, COM then grows on some outer stone surfaces as cements (COM_CE_; lime green layers in Supplementary Fig. 1c, labeled COM_CE_ in Fig. 2c and Supplementary Fig. 4a,b), which exhibit crystal-face specific differences in organic matter concentrations (*sector zoning*) (labeled SZ in Fig. 2c and Supplementary Fig. 4b)^26^. Uric acid cement crystals (UA_CE_) then grow on outer surfaces of the stone complex (gray layers in Supplementary Fig. 1c, labeled UA_CE_ in Fig. 2c and Supplementary Fig. 4a,b). Other outermost COM_C_ stone surfaces continue to dissolve and are replaced by COD and COM (COD_R_ and COM_R_, cyan in Supplementary Figs. 1c, 4c–f), which is consistent with previously observed “intimate COD and COM relationships^6^”.

COD_FF_ aggregates, which reach 300 µm in diameter (dark blue S3 in Supplementary Fig. 1c), are consistent with previous observations of COD morphology within kidney stones and likely form under hypercalciuric conditions^27^. Their large size requires that crystals aggregate in urine collected in the renal calyx and pelvis as opposed to filtrate in the nephron collecting ducts, which only reach ~150 µm in diameter^12,15^. These large COD_FF_ aggregates form the nucleus (*nidus*) that COM_C_ encrusts (Fig. 1c). The symmetry of the COM_C_ concentric layering indicates free-floating crystallization while completely bathed in urine. Growth while attached to tissue on one side would create discontinuous and asymmetric COM_C_ layering around the COD_FF_ nidus^12,15^. In addition, COM_FF_ are observed to have landed on the growing surfaces of COM_C_. These crystals become entombed in, and encrusted by, the concentric COM_C_ nano-layering (Figs. 1c and 2, Supplementary Fig. 4c,d) and are called protrusions^28^ into COM_C_.

The HSE (Fig. 1) of this representative stone has broad implications for understanding the growth history of other types of kidney stones, including those that originate in the renal papilla as interstitial deposits of calcium phosphate (*apatite*) called Randall’s plaques, or in the ducts of Bellini called Randall’s plugs^29–31^. Randall’s plaque commonly erupt into the renal calyx and pelvis, or are released through the nephron, and begin to accrete COD_FF_ and eventually COM_C_ (HSE 4) while bathed in urine^12^. Instead of the initial stages of apatite nidus growth^32^, CaOx kidney stones in the present study have a nidus composed of free-floating COD_FF_ and COM_FF_ (HSE 1–3, Fig. 1). The lack of apatite crystal dissolution fabrics within COD_FF_ and COM_C_ in the MP-series stone fragments implies that these CaOx stone fragments likely did not contain a precursor Randall’s plaque or plug nidus.

### Kidney stones dissolve *In vivo*

Results from the current study modify the long-held working assumption that COM is strongly insoluble *in vivo*^3,12,15^, except perhaps within some individual cell organelles (*phagolysosomes*)^33^. In addition, although the entombment of organic matter has been previously documented^12,15^, the clinical importance of these biomass-rich nano-layers remains undetermined^28^. Our results indicate that kidney stones repeatedly dissolve *in vivo* (Figs. 1 and 2, Supplementary Figs. 1–4; summarized as dissolution of COD_FF_ outer surfaces [HSE 3], dissolution of COD_FF_, COM_FF_ and COM_C_ via four types of cross-cutting crystal fabrics [HSE 5], and mimetic replacement of COM_C_ by COM_M_ [HSE 6]) and highlight the intrinsic relationship between organic-rich and mineral-rich nano-layering.

The repeated dissolution, crystallization and resultant remodeling of crystalline architecture that takes place during CaOx stone growth is analogous to the commonly observed post-depositional physical, chemical and biological alteration observed in natural mineral deposits (*diagenesis*)^24^. The sector zoning in COM_CE_ indicates that these crystals may be more soluble and thus more susceptible to diagenesis than expected with respect to calculated urine supersaturation states^25^, as controlled by the differential entrapment of organic matter on specific COM crystal faces^34,35^. The biomolecules present within the human kidney may also play a major role in driving the multiple events of dissolution as recorded in the HSE (Fig. 1). These are normal constituents of urine chemical composition^36^, and could plausibly include biomolecules derived from a resident microbial community (*microbiome*)^13^. However, the composition and potential effects of these biomolecules are currently unknown^37^.

### COM_C_ nano-layers

We use fast Fourier transform (FFT) frequency analyses to compare the nano-layering within individual COM_C_, COD_FF_ and COM_R_ (Supplementary Fig. 5). Both COM_C_ and COD_FF_ exhibit highest frequencies and thinnest nano-layers (Supplementary Fig. 5a–d,g–j). The crystalline architecture of COM_C_, COD_FF_ and COM_R_ exhibit different patterns in three distinct optical modalities applied (POLPC, CPOL, SRAF, Supplementary Fig. 11) indicating that multi-modal approaches are required to investigate kidney stone crystalline fabrics (Supplementary Fig. 5). Nano-layering is the most volumetrically dominant component of COM_C_ (Figs. 1 and 2) and occurs in well-defined organic matter- and mineral-rich couplets^12,15,38^. However, a comprehensive understanding of the mechanisms controlling the abrupt switch between deposition of each organic matter-rich and mineral-rich nano-layer is unknown^9,28,39,40^. Under SRAF imaging, bright AF indicates organic matter-rich layers, while dim AF represents the adjacent mineral-rich couplet layers (Fig. 2d,e and Supplementary Figs. 5 and 12). In contrast, when observed under transmitted-light polarization and phase contrast (CPOLPC), the brighter layers are crystal-rich and the dimmer layers are organic matter-rich (Fig. 2f,g). While SRAF imaging reveals sharp well-defined COM_C_ layer couplets at a spatial resolution as fine as ~140 nm (Fig. 2e), previous transmission electron microscopy imaging of other kidney stones has detected even finer layering at ~50 nm in thickness^41^. If these ~50 nm-thick layers are present in the six Mayo Clinic patient stones, they would not have been optically resolved with SRAF in the present study, but instead, would optically average into ~140 nm-thick layers within each couplet. As a result, the actual number of COM_C_ layer couplets and their frequencies in the present study could be a 2–3-fold underestimate. Given these detection limits, the observed COM_C_ is composed of ~140–250 nm couplets based on the optical resolution of our microscope system.

An initial interpretation of these COM_C_ couplets (Fig. 2d–g) is that the organic matter-rich nano-layers are films of biomolecules (peptides, proteins, etc.) entombed between mineral-rich nano-layers^28^. Potential reasons for these rapid nano-layer shifts might include frequent changes in human host and kidney physiology, urine biochemistry and perhaps even microbiome ecology and activity. Alternatively, these layers are also comparable to the oscillatory zoning found in many minerals, where it is believed that a kinetic feedback mechanism results in periodic oscillations of crystal growth and impurity occlusion independent of biological activity^42^. Further analysis of the composition and concentration of organic matter entrapped in each cortex nano-layer will be required to establish a mechanistic hypothesis for their deposition. Since the exact amount of time required to form any given kidney stone is difficult to constrain, it is uncertain precisely how long it takes to deposit each nano-layer couplet. However, previously published observational data regarding how long it takes CaOx stones to grow^43,44^ implies that several thousands of nano-layers could possibly form within weeks or months. Given these rough estimates, each nano-layer may have formed on a sub-daily basis of hours or in some cases even minutes. If correct, kidney stones could be “read” in the future under clinical conditions as an unprecedented ultrahigh-sensitivity record of *in vivo* human renal function and dynamic biogeochemical reactions.

### Biomineralization in natural and engineered environments

The alternating organic matter- and mineral-rich nano-layers comprising COM_C_ are strikingly similar to those seen in other modern and ancient sedimentary deposits. These include marine stromatolites, ooids and oyster shells and pearls, as well as terrestrial hot-spring travertines and cave speleothems, among many other deposits^45–47^. Previous geobiology studies of these natural deposits have only partially deciphered the relative influence of the physical, chemical and biological factors that are active at the time of layered deposition. COM_C_ nano-layer couplets represent a previously unknown template for understanding the mechanisms that fundamentally control shifts between biotic and abiotic processes during biomineralization. These mechanisms are directly applicable to understanding biomineralized deposits common to other natural and engineered environments in fields that range from environmental sustainability and energy production, to medical discovery and space exploration.

BF microscopy with a theoretical optical resolution of approximately 1-μm was used to compare the nano-layer couplets in kidney stones (Supplementary Fig. 6a–c,j) with travertine formed within ancient Roman aqueducts^45,47^ (Supplementary Fig. 6d–f,k) and cave limestone deposits (*speleothems*, Supplementary Fig. 6g–i,l)^45,46^. Out of necessity, these analyses are completed at a micrometer-scale resolution instead of the nanometer-scale since the cave and aqueduct systems have orders-of-magnitude higher crystal growth rates than those in the kidney (Supplementary Fig. 13). These higher rates of crystal growth dramatically increase the thickness of each layer, which makes only one or two layers fill an entire frame at super-resolution, making them incompatible for frequency analysis (Supplementary Fig. 13a–d). Requirement of lower magnification imaging indicates that CaOx kidney stone layers are 10-times higher frequency (~1.6 µm/layer) than those in the cave deposits (~16 µm/layer), and 3-times higher frequency than those in the aqueduct travertine (~5 µm/layer; Supplementary Fig. 6m). Although not yet proven, the significantly thinner and higher frequency nano-layers in kidney stones (Supplementary Fig. 6) may be the result of the short-time scales over which human renal function and biochemistry can change (i.e., seconds to hours). This may also reflect the abundant and diverse sources of inhibitors in the renal environment compared to those in other geological and engineered settings. In addition, the ~140 nm-scale and finer nano-layering in CaOx kidney stones is significantly smaller than the µm-size of whole microbial cells and their associated microbial mats that directly influence layering in other geological deposits such as stromatolites^45,48^. As a result of these size constraints, kidney stone biomineralization must be controlled by some combination of human host and/or kidney microbiome derived biomolecules rather than whole cells.

CaOx kidney stones occur throughout the animal kingdom^49^. While euhedral CaOx crystals similar to COD_FF_ and COM_FF_ are also common in plants, no COM_C_ has yet been reported^50^. This lack of COM_C_ in plants likely reflects the absence of the type of high flow-through hydrodynamic environment present in the kidney, which is required to consistently deliver dissolved ions to the site of COM_C_ crystallization. CaOx crystallization in terrestrial plants serves to store carbon and H_2_O for later use in times of reduced carbon availability and drought^50,51^. By analogy, it is also possible that COD_FF_, COM_FF_ and even COM_C_ crystallization in animals may serve to store water for later use when the kidney ecosystem is stressed. *In vitro* batch reactor experiments^52^, as well as our own microfluidics experimentation (Supplementary Figs. 7 and 17) to test CaOx growth dynamics, have successfully grown free-floating polymorphic COD_FF_, COM_FF_ and their aggregates (equivalent to HSE1, Supplementary Fig. 8). While COD-to-COM and apatite-to-COM conversion has been demonstrated convincingly, no previous experimentation has grown COM_C_ with nano-layer couplets^3,8,53,54^.

### Clinical and future implications

Human beings almost constantly create urine supersaturated with respect to CaOx, yet the question remains as to why only 1 in 11 people actually grow symptomatic kidney stones^12,15^. Our study illustrates that understanding kidney stone growth requires knowledge of not only urine chemistry, but also crystalline architecture and chemistry of the stones, as well as the biomolecules derived from the host animal, kidney itself and possibly resident microbes. The HSE (Fig. 1a,b) is a new synthesis that identifies an unexpected roadmap of therapeutic targets for prevention and treatment, which include: 1) prevent COD_FF_ and COM_FF_ aggregation and their twinning; 2) promote and maintain COD_FF_ growth, continue COD_FF_ surface dissolution, then completely dissolve COD_FF_ prior to COM_C_ encrustation; 3) periodically induce COD_FF_ growth during COM_C_ formation to create layers that can be dissolved; 4) enhance any of the later-stage HSE dissolution events; and 5) disrupt crystal, urine and biomolecule components that combine to control the switching between organic matter-rich and crystal-rich COM_C_ nano-layers. All of these therapeutic targets would involve the introduction of macromolecules that either inhibit or promote crystal growth and dissolution, and lead to less crystal aggregation. The development of tools to read the crystalline architecture of kidney stones in a clinical setting would allow rapid evaluation and determination of which of these interventions to pursue. It has been confirmed for over a decade that precursor forms of amorphous CaOx exist as they do in calcium carbonate and calcium phosphate biomineralization^55–57^. Through a multi-step process, these amorphous CaOx phases fully transform into CaOx crystals. Although they leave behind no obvious record of their existence, clinical interventions targeting these early upstream events of biomineralization should also be considered for the treatment of kidney stones to target these transient states to destabilize the forming crystals. Taken together, these geobiology approaches will also have a transformative impact on the study of gallstones, atherosclerosis, osteoporosis and a variety of other human biomineralization conditions that involve multiple events of crystal growth and dissolution.

## Methods

### Detailed methods are provided online in Supplementary Materials

#### Study approval

All methods reported in this manuscript were carried out in accordance with the basic medical research study, which was reviewed and approved by the Mayo Clinic Institutional Review Board (IRB 09-002083), and the outcomes of this study will positively affect the future management of kidney stone formers. Written informed consent was obtained from all six Mayo Clinic patient participants.

#### Kidney stone collection and thin sectioning

Kidney stones were collected and analyzed from all six patients by a single urologist using standard percutaneous nephrolithotomy (PCNL) procedures under sterile operating room conditions at the Mayo Clinic in Rochester, Minnesota. A line of section for thin sectioning for each stone was carefully selected under a Zeiss Axio Zoom.V16 Microscope (Carl Zeiss, Oberkochen, Germany), along the orientation that would exhibit a complete cross-section of earliest-to-latest crystalline stone growth. Photographs and descriptions were sent with the six stones to Wagner Petrographic Ltd. in Linden, Utah. Here they prepared standard-sized (24 mm × 46 mm), uncovered (no cover slip), doubly polished thin sections. Stones were first vacuum impregnated and then mounted on borate silicate glass slides with clear low-viscosity cathodoluminescence-resistant epoxy impregnation to prepare double-sided polished, 20 µm-thick petrographic thin sections.

#### Thin section imaging

Thin sections were imaged on a wide variety of optical modalities (Supplementary Fig. 11). A Zeiss Axio Observer system (Carl Zeiss, Oberkochen, Germany) with a Zeiss Axiocam 512 MRc was used to capture the BF, POL, PC, POLPC, CPOL, CPOLPC and WAF images across a broad range of magnifications (10x: 0.3 NA; 20x: 0.8 NA; 63x: 1.4 NA; and 100x: 1.46 NA). The objectives used were Plan-Neofluar (10x), Plan-Apochromat (20–63x) and alpha Plan-Apochromat (1.46 NA). WAF was acquired in three channels, including DAPI, FITC and Rhodamine filters. The confocal auto-fluorescence and Airyscan super-resolution nano-layers observed in the samples were investigated and quantified using a Zeiss LSM 880 Laser Scanning Microscope with Airyscan Super-Resolution. A spectral confocal system (Zeiss LSM 710) with a spectral PMT detector AF emissions and a TPAF spectral imaging, a secondary complementary FLIM technique (Fast FLIM, ISS, Champaign, IL) were used to distinguish the AF emissions produced by organic matter trapped within the calcium oxalate crystals, from those produced by the epoxy required to impregnate and mount the stones. All images were processed using the Zeiss Zen Blue and/or Black software to display either minimum and maximum or best-fit properties unless otherwise stated in the figure legends. In addition, red-green-blue (RGB) curves were adjusted individually or together to highlight all the crystal intensities in individual frames across the whole specimen. Where required, a non-linear gamma correction of 0.45 or 0.70 was applied to enhance faint AF crystal intensities in the same Zen program under the spline display mode property and all other corrections are presented in the corresponding figure legends themselves. Final images were cropped, resized and assembled using Adobe Photoshop (Adobe Systems Inc., San Jose, CA) to fit the required format. Adjustment and correction models for all images are reported in Supplementary Figs. 18–22.

#### Elemental analysis of kidney stones

Elemental analysis of kidney stones was performed under standard Mayo Clinic infra-red spectral analyses protocol. To qualitatively determine chemical composition, samples were analyzed with energy dispersive x-ray spectroscopy (EDXS) using an FEI Quanta FEG 450 FESEM (Hillsboro, OR).

#### *In Vitro* COM and COD crystal formation in a microfluidic device

A pilot study of initial kidney stone nidus crystal growth (HSE 1, Fig. 1) was conducted using a silica microfluidic device designed with a pore-structure design that mimics the renal calyx and pelvis environments. A solution of 0.5% (w/v) of kidney stone particles and urine was prepared and injected into the micromodel at a flowrate of 100 µl/h for 24 hrs. After this, a laboratory urine solution containing both calcium (Ca^2+^) and oxalate (C_2_O_4_^2−^) concentrations of 1 mM each (pH of 7.2) was prepared and injected into the micromodel at a rate of 100 µL/h to promote crystal formation in the micromodel. CaOx crystal growth was tracked with a Nikon Eclipse Ti-E epi-fluorescent inverted microscope with an Andor Zyla color camera attachment. Mineralogy of the COM and COD crystals that precipitated within in the micromodel was confirmed with Raman Spectroscopy (LabRam HR Evolution NIR, Horiba Scientific) collected between 0–1600 cm^−1^ with a 532 nm DPSS laser and confirmed with the RRUFF online database.

## Electronic supplementary material

Supplementary Materials

Supplementary Video 1

Supplementary Video 2

## Data Availability

All raw data images from the microscope and point-by-point Excel spreadsheet data for FFT and line tracing graphs can be downloaded from the open access website https://figshare.com from the following links: Main Figures Raw Data Link: https://figshare.com/s/d130f7175e0266a62b97. Supplementary Raw Files Link: https://figshare.com/s/9d68faada43222c96a52.
