# Supplementary material for: Geobiology reveals how human kidney stones dissolve in vivo
Source: Sci Rep. 2018 Sep 13;8:13731. doi: 10.1038/s41598-018-31890-9 (PMC6137216; doi:10.1038/s41598-018-31890-9)
Supplement: Supplementary file 1 — Supplementary Materials [file 41598_2018_31890_MOESM1_ESM.docx]

# Geobiology reveals how human kidney stones dissolve *in vivo*

Mayandi Sivaguru, Jessica J. Saw, James C. Williams, Jr., John C. Lieske, Amy E. Krambeck, Michael F. Romero, Nicholas Chia, Andrew L. Schwaderer, Reinaldo E. Alcalde, William J. Bruce, Derek E. Wildman, Glenn A. Fried, Charles J. Werth, Richard J. Reeder, Peter M. Yau, Robert A. Sanford and Bruce W. Fouke

# Supplementary Materials

1. **Supplementary Extended Methods (p. 1-7)**
2. **Supplementary References (p. 8)**
3. **Supplementary Figures (p. 9-30)**
4. **Supplementary Videos Captions (p. 31)**

**I. Supplementary extended methods:**

## Permits and IRB Approval. This basic medical research study was reviewed and approved by the Mayo Clinic Institutional Review Board (IRB 09-002083), and the outcomes of this study will positively affect the future management of kidney stone formers. Written informed consent was obtained from all six Mayo patient participants.

## Mayo Patient Medical Histories. The medical records of all patients were reviewed for pertinent historical and diagnostic data. This six-patient Mayo Clinic cohort represented, in general, the profile of risk factors and comorbidities expected in a cohort of recurrent calcium oxalate stone formers. The patients ranged in age from 42 to 85 years-old and were a mixed gender (4 female and 2 male) group of Caucasians with no previous family history of stone formation. All patients were either overweight or obese (mean BMI 32 kg/m2, normal range BMI = 19-25 kg/m2). Two of them had medically controlled hyperlipidemia (high cholesterol) and hypertension (high blood pressure). Only two patients were diagnosed with diabetes mellitus type II, while most of the patients had high blood glucose levels (average 125.4 mg/dL). Creatinine and blood urea nitrogen (BUN) levels were generally within a range considered to be normal, indicating healthy renal function in all six patients. Their urine also contained citrate, sulfate, and magnesium stone inhibitors at concentrations considered to be normal. These patients’ urine also exhibited a higher-than- average level of urinary supersaturation for CaOx (2.3 DG, reference mean = 1.77 DG). The patients all had increased 24-hour urine calcium excretions (353.4 mg/24-hr, normalmale < 250 mg/24-hr, normalfemale < 200 mg/24-hr), while their urine oxalate excretions were within the reference range.

## Kidney Stone Collection and Handling. Kidney stones were collected and analyzed from all six patients by a single urologist (author AEK) using standard percutaneous nephrolithotomy (PCNL) procedures58 under sterile operating room conditions at the Mayo Clinic in Rochester, Minnesota. Each patient had multiple small (0.5 to 2 mm-diameter) and irregularly shaped kidney stone fragments (Supplementary Figure 9). These were removed using sterile forceps and placed directly into sterile screw-top 25 ml Falcon conical centrifuge tubes. Each tube was then immediately placed within a -80oC Taylor-Wharton CX Series dry shipper dewar (Borehamwood, UK), and shipped to the BSL2-certified geobiology laboratory in the Carl R. Woese Institute for Genomic Biology (IGB) at the University of Illinois at Urbana-Champaign. For each patient, a single kidney stone fragment was removed using sterile forceps and placed into a separate Falcon tube for petrographic thin section analysis. The mineralogical composition of the six stones was estimated with IR analyses, which indicated that they were all primarily composed of calcium oxalate, in the forms of COD and COM (Supplementary Figure 9). In particular, the MP2 stone exhibited a 100% COM in the IR analysis at the Mayo Clinic.

## Roman Aqueduct and Cave Speleothem Sample Locations and Collection. A 65 cm-long speleothem CaCO3 stalagmite was collected in Summer 2002 from Fogelpole Cave46 located 50 km southeast of St. Louis, Missouri (N 38o 11’ 49.19” , W 90o 7’ 45.41). A 10 cm-thick sample of CaCO3 travertine was collected in Summer 2012 from the basal channel of the Anio Novus aqueduct in Romavecchia47 located 15 km southeast of downtown Rome, Italy (N 41°53'32.867″, E 12°29'7.166″). Detailed descriptions of sample sites and compositions for both the cave speleothem and aqueduct travertine samples are previously published45-47. Vertical cross-sectional slabs of both samples were cut on a diamond-impregnated saw, polished and cut again into 3cm x 2cm x 1cm billets. Each billet was then impregnated with blue epoxy, polished, mounted onto standard-sized petrographic glass slides, and cut and polished into 20-30 mm-thick wafers at Wagner Petrographic, Linden, Utah.

## Thin Sectioning of Kidney Stones. One kidney stone from each of the six Mayo patients was rinsed in deionized water, dried, evaluated and oriented for thin sectioning under a clean laminar flow hood. A line of section for thin sectioning was carefully selected under a Zeiss Axio Zoom.V16 Microscope (Carl Zeiss, Oberkochen, Germany), along which to orient a 20-30 µm-thick rock wafer that would exhibit a complete cross-section of earliest-to-latest crystalline stone growth (Supplementary Figures 9a, d, g, j, m, p). Photographs and descriptions were sent with the six stones to Wagner Petrographic Ltd. in Linden, Utah. Here they prepared standard-sized (24mm x 46 mm), uncovered (no cover slip), doubly polished thin sections. Stones were first vacuum impregnated and then mounted on borate silicate glass slides with clear low-viscosity cathodoluminescence-resistant epoxy impregnation to prepare double-sided polish, 20 µm-thick petrographic thin sections. Upon their return to Illinois, an image of the entire kidney stone thin section was taken on a Zeiss Axio Zoom.V16 microscope with a 1.0x Plan Apochromat NA 0.25 objective (Supplementary Figures 6c, f, i and 9b- c, e-f, h-i, k-l, n-o, q-r). The samples were illuminated with a DL 450 LED light source base, and imaged with a Zeiss Axiocam 512 color camera (Carl Zeiss, Oberkochen, Germany).

## Bright-Field (BF), Polarization (POL), Phase Contrast (PC), Polarization Phase Contrast (POLPC), Circular POL (CPOL), Circular POLPC (CPOLPC) and Wide-Field Auto- Fluorescence (WAF). A wide variety of optical modalities (Supplementary Figure 11) were applied in the present study to image the kidney stone thin sections16,17,59-65. These included BF, POL, PC, POLPC, CPOL, CPOLPC and WAF across a broad range of magnifications (10x: 0.3 NA; 20x: 0.8 NA; 63x: 1.4 NA; and 100x: 1.46 NA). The objectives used were Plan-Neofluar (10x), Plan-Apochromat (20- 63x) and alpha Plan-Apochromat (1.46 NA). WAF was acquired in three channels, including DAPI, FITC and Rhodamine filters. All optical and electron microscopy imaging presented in this study was conducted on instruments housed in the Microscopy and Imaging Core Facility of the Carl R. Woese Institute for Genomic Biology on the University of Illinois Urbana- Champaign campus. This was the first North American laboratory selected as a Zeiss Labs@Location Partner by Carl Zeiss LLC, a recognition that facilitated the integrated assemblage of 12 microscopy modalities applied for the first time in the present study.

A Zeiss Axio Observer system (Carl Zeiss, Oberkochen, Germany) with a Zeiss Axiocam 512 MRc was used to capture the images as described previously for multiple modalities17. The POLPC technique places a phase ring in the path of the light to reject 0-order rays, which enhances the resolution and contrast of the image. This is particularly useful when imaging the cortex of the kidney stones compared to conventional POL technique. This optical path modification permitted higher frequency cortex layering to be imaged using POLPC (Supplementary Figures11b-e). This technique was further modified with circular polarizers in CPOLPC instead of the conventional crossed polarizers in POLPC, permitting all crystals illuminated irrespective of their axis of orientation to produce an isotropic image (Supplementary Figures 11f-i). A comparison between conventional POL, POLPC, CPOL, and CPOLPC are shown in Supplementary Figure 11.

**Confocal Auto-Fluorescence (CAF) and Airyscan Super-Resolution AF (SRAF).** The dark-light nano-layering (organic matter-rich layers and mineral-rich layers) observed in the six calcium oxalate Mayo patient kidney stones is significantly greater than that observed in other natural and engineered environments. This layering was investigated and quantified using a Zeiss LSM 880 Laser Scanning Microscope with Airyscan Super-Resolution65. This instrument has a resolution significantly greater than all other currently available confocal diffraction- limited microscopy techniques by a factor of 1.7, to provide a resolution of ~140 nm16,62,64,65. We have used this system in both SRAF (Figures 1c, 2b-e, Supplementary Figures 1b, 2a-d, f-g, 3b-e, 4a-d, 5b-f, 8b-d, 12a-c) and CAF (Fig. 2a, Supplementary Figures 8e, g) modes. Excitation and emission wavelengths that were collected include: 405 nm excitation (emission collected between 410-460 nm), 488 nm excitation (emission collected between 500-550nm) and 561 nm excitation (emission collected between 570-615 nm). An internal photo-multiplier tube detector was used to scan entire stone thin sections using a 63x Plan Apochromat (NA 1.4) or 100x Alpha-Plan Apochromat (NA 1.46) oil immersion objective in regular confocal mode with the built-in tile-scan function. At the same time, a transmitted-light image was obtained using a 561 nm laser transmission photomultiplier (PMT) detector on the same system. A complete image of the entire 4 x 4 mm MP2 stone thin section was collected at 145 nm-resolution per pixel in XY. To cover the entire stone at high resolution, a total of 361 individual field of view images (each comprising 1,332 x 1,332 pixels; ~193 x 193 µm) were combined by tiling the entire stone in 19 x 19 tile regions in four separate channels. This represents a total of 1,444 images, covering a range of 25,308 x 25,308 pixels (3,662 x 3,662 µm) in two-dimensions (2D). For the other five patient stones, images were collected in the same three AF channels, only at specific locations of interest (the entire stone is not tiled as in the case of the exemplar stone MP2, Supplementary Figure 10). However, at some locations the other stones including MP2 are tiled under much smaller area at a higher resolution, each comprising a total of 16 individual field of view images (1,332 x1,332 pixels; ~193 x93 µm each) covering the region in 4 x 4 tiles in four separate channels. This covers an area of 764 x 764 µm at a resolution of 140 nm (XY=0.037 nm) accomplished by using the zoom optics of the Airyscan system. For the imaging of all six Mayo patient stones, the main beam splitters (MBS) used in the light path of the detector were MBS 488/561/633 for visible and MBS 405 for invisible light paths. Raw data images from 32 Airyscan detectors were processed using the Airyscan processing modality using the auto-2D or 3D mode (for Z stacks) to obtain a super-resolution image.

## Two-Photon Auto-Fluorescence (TPAF) Microscopy with Spectral Detection. Next, it was necessary to distinguish the AF emissions produced by organic matter trapped within the calcium oxalate crystals, which comprise the kidney stones from the AF emissions produced by the epoxy required to impregnate and mount the stones (Supplementary Figure 15). To do this, a spectral confocal system (Zeiss LSM 710) with a spectral PMT detector was used to separate spectral bands that were ~10 nm apart over the entire visible spectrum60. A 780 nm two- photon excitation was used to characterize the spectral signatures from 500-720 nm with a short- pass dichroic mirror (Supplementary Figure 15a). The spectral signals were decoupled using a spectral un-mixing algorithm using the built-in Zeiss Zen Black version 2.3 software. This image analysis program rejects epoxy-sourced emissions by setting them as a background signal, which is removed, leaving only organic matter-derived emissions in the final image (Supplementary Figure 15b). These respective organic matter and epoxy emission profiles were plotted against the entire acquired wavelength range (Supplementary Figure 15c). We have also tested kidney stones embedded but not infiltrated with epoxy as well as without polishing using grits, which are typical sources of AF. Both of these stones exhibited consistent concentric layering in all three fluorescent channels (Supplementary Figure 16).

## Fluorescence Lifetime Imaging Microscopy (FLIM). In addition to the TPAF spectral imaging, a second complementary FLIM technique59 (Fast FLIM, ISS, Champaign, IL) was used to further separate organic matter from epoxy emissions. The FLIM module was custom coupled with the aforementioned Zeiss LSM 710 system at the non-de-scanned detector port, which provided yet another independent confirmation of the presence of organic matter within the kidney stones’ crystalline architecture. In addition to capturing the emission photons in confocal microscopy, the decay time of emission photons was measured in the frequency domain. This emission lifetime of photons is unique for any specific fluorescing molecule59, and therefore organic matter can be differentiated from the epoxy. Each FLIM image was collected from precisely the same position where the TPAF spectral data was collected to permit accurate spatial comparison. The built-in VistaVision software within the FLIM system was used to control photon collection using an avalanche photodiode as a detector with a 256 x 256 pixel array from an 80 MHz 2-photon light source with a pixel dwell time of 6.3 microseconds. An average of 100 frames were collected after calibrating the system with Rhodamine 6G, which has a fluorescence lifetime of 4.0. The signals were integrated through a red emission filter (565-615 nm). Finally, the counts were analyzed under frame average mode and the correlation between the modulation and phase was found while analyzing the lifetime. The lifetime data, plotted on a phasor plot, identified the pure and mixed components of organic matter and epoxy (Supplementary Figures 15d-j).

## Fourier Frequency Analysis of layering in COMC, CODFF, COMR and natural deposits. We analyze the COMC, CODFF and COMR nano-layering frequencies using the SRAF images (Supplementary Figure 5). Using the same SRAF images, we compare the organic matter AF using the line intensity profiles in the native Zeiss Zen Blue software. We also compare the dark-light nano-layering in CaOx kidney stones with the layering observed in travertine deposited within Roman aqueducts47 and cave limestone (speleothem) deposited in Illinois Caverns (Supplementary Figure 6)46. Thin sections of the travertine and speleothems were prepared as described previously for the kidney stones. These were imaged under the Zeiss Axio Zoom.V16 Microscope at the same magnification and pixel resolution used for the kidney stone thin sections. These images were cropped, converted to 8-bit grey scale, and processed for fast Fourier transformation (FFT) using ImageJ (an open source image analysis software)66. Line profile analyses of FFT image spectra were completed in the same program using plot profile module. Each line profile (25 or 30 pixels wide) was constructed from the center pixel of the Fourier spectrum, to the maximum frequency distribution angle up to the edge of the image. The intensity distribution (Y-axis) shows the frequency plotted against the retrieved resolution (cycles/micron in the X-axis), which was used for comparison. The equation to calculate cycles/micron is based on the pixel dimension and resolution of each pixel as follows: (pixel size in XY x image dimension in Y) / (pixel number x 1000) as previously described61 (raw excel data with equations for this calculation is provided online as point-by-point data for all plots showed in this manuscript). In addition, the actual grey-scale FFT look-up tables were converted to a ‘Spectrum’ look up table (LUT) in the Image J program to highlight the frequency distribution. Similar FFT analyses of original dark-light nano-layers were analyzed from SRAF images after combining all three channels and making a single grey-scale image to understand the differences in frequencies of layering among individual calcium oxalate crystals (COMC, CODFF and COMR, Supplementary Figure 5).

## Elemental Analysis of Kidney Stones using Field Emission Scanning Electron Microscopy (FESEM) Coupled with Energy Dispersive Spectroscopy. Bulk-rock compositional analyses were performed on all six of the patient kidney stones using standard Mayo protocols. Briefly, stone fragments were first crushed into a fine powder and then mixed with potassium bromide for infra-red (IR) spectral analyses and compared to their reference spectra67. To qualitatively determine the chemical composition to the MP2 and MP9 stone fragments (analysis locations are presented in Supplementary Figure 17), samples underwent energy dispersive x-ray spectroscopy (EDXS) using an FEI Quanta FEG 450 FESEM (Hillsboro, OR). The ultra-thin 20-30 µm-thick sections of the kidney stones were sputter coated for 70 s with a gold palladium (Au/Pd) target using a Denton DESK II TSC sputter coater (Denton Vacuum, Turbo Sputter Coater, Moorestown, NJ). The sample was then loaded together with seven standards of pure crystal powder standards of calcium oxalate monohydrate, uric acid, L- aspartic acid, calcium carbonate, ammonium-magnesium phosphate hexahydrate, L-cystine, L- glutamic acid. Standard settings were used for the analyses, including a 20 kV beam with a spot size of 4.0, working distance of ~10 mm, and 250x magnification with a dwell time of 300 ns.

## Image Adjustments, Analysis, Preparation and Presentation. All images were processed using the Zeiss Zen Blue and/or Black software to display either minimum and maximum or best-fit properties68 unless otherwise stated in the figure legends. All raw data was stored in native CZI or LSM 5 format and then exported as TIF files with no compression after adjusting the display properties using the same program. Final images are cropped (in Adobe Photoshop) to remove the excess epoxy from around both brightfield and confocal fluorescence images for clarity. In addition, red-green-blue (RGB) curves were adjusted individually or together to highlight all the crystal intensities in individual frames across the whole specimen. Where required, a non-linear gamma correction of 0.45 or 0.70 was applied to enhance faint AF crystal intensities in the same Zen program under the spline display mode and such corrections are presented in the corresponding figure legends itself. Final images were assembled using Adobe Photoshop (Adobe Systems Inc., San Jose, CA), after cropping and resizing to fit the required format. All adjustment and correction models for figures are reported in Supplementary Figures 18-22.

## In Vitro COM and COD Crystal Formation in a Microfluidic Device. A pilot study of initial kidney stone nidus crystal growth (HSE 1, Fig. 1) was conducted using a silica microfluidic device designed with a pore-structure design that mimics the renal calyx and pelvis environments. The micromodel dimensions are 2 cm x 1 cm with a depth of 20 µm. Cylindrical pillars 300 µm in diameter create 40 µm-diameter pores. The microfluidic device was fabricated using standard photolithography and inductively coupled plasma deep reactive ion etching63. PEEK and ETFE fittings and tubing were used to both deliver and remove fluid from the micromodel. Plastic syringes (BD Disposable Syringes with Luer-Lok, 5mL) and a microfluidic syringe pump (Cole-Parmer) controlled the injection of solutions into the micromodel.

CaOx kidney stone fragments were first crushed with an agate mortar and pestle, then passed through a stainless steel 20 µm mesh sieve. Kidney stone particles 20 µm in diameter or less were selected of the size limitations by the size and design of the micromodel. A 250 mL urine sample was collected and immediately filtered through a 0.22 µm PES membrane. A stock solution of oxalic acid (HO2CCO2H; Sigma-Aldrich; reagent-plus, ≥99%) was prepared at 0.5 M, with the pH then adjusted to 7 by addition of NaOH. A stock solution of calcium chloride (CaCl2•2H2O; Sigma-Aldrich; ACS reagent, ≥99%) was prepared at 0.5 M.

Prior to the experimental run, the micromodel was saturated and disinfected by injecting a solution of 70% EtOH at 100 µL/hr for 24hrs. Autoclaved distilled water was then injected and flowed through for 2 hours at 100 µL/hr to purge the EtOH. This was followed by the injection of sterile filtered urine for 2 hrs at 100 µL/hr to purge H2O and set the initial conditions for the experiment. A solution of 0.5% (w/v) of kidney stone particles and urine was prepared and injected into the micromodel at a flowrate of 100 µl/hr for 24 hrs. This was done to allow the powdered kidney stone particles to move into and attach within the pore-network and serve as nucleation sites for future COM and COD crystal formation. After this, a laboratory urine solution containing both calcium (Ca2+) and oxalate (C2O42-) concentrations of 1 mM each (pH of 7.2) was prepared and injected into the micromodel at a rate of 100 µL/hr to promote crystal formation in the micromodel. Note that these concentrations of calcium and oxalate are approximately 20x the thermodynamic solubility product (Ksp) value (i.e., 0.052 mM at 25˚C). Trial and error runs at concentrations ranging from 0.052 to 0.52 mM did not result in CaOx nucleation. Conversely, robust crystal growth was observed in the micromodel within 24 hrs window when a freshly prepared Ca2+ + C2O42- urine solution was introduced. After 48 hrs of introducing the fresh urine solution, CaOx precipitates were observed in the syringes as well. This implies that dissolved Ca2+ and C2O42- remained in solution that was retained within the syringes after the urine fell below Ksp saturation values and thus no further precipitation occurred within the micromodel. CaOx crystal growth was tracked with a Nikon Eclipse Ti-E epi- fluorescent inverted microscope with an Andor Zyla color camera attachment. Mineralogy of the COM and COD crystals that precipitated within in the micromodel was confirmed with Raman Spectroscopy (LabRam HR Evolution NIR, Horiba Scientific) collected between 0-1600 cm-1 with a 532 nm DPSS laser and confirmed with the RRUFF online database67 (SupplementaryFigure 14).

# II. Supplementary References

1. Patil, A. V. A novel 5-part Percutaneous Access Needle with Glidewire technique (5- PANG) for percutaneous nephrolithotomy: our initial experience. *Urology* **75**, 1206- 1208, doi:10.1016/j.urology.2009.11.027 (2010).
2. Sivaguru, M. *et al.* Imaging horse tendons using multimodal 2-photon microscopy. *Methods* **66**, 256-267, doi:10.1016/j.ymeth.2013.07.016 (2014).
3. Sivaguru, M., Fried, G. A., Miller, C. A. H. & Fouke, B. W. Multimodal Optical Microscopy Methods Reveal Polyp Tissue Morphology and Structure in Caribbean Reef Building Corals. *Jove-J Vis Exp*, doi:ARTN e51824 10.3791/51824 (2014).
4. Sivaguru, M. *et al.* Application of an advanced maximum likelihood estimation restoration method for enhanced-resolution and contrast in second-harmonic generation microscopy. *J Microsc* **267**, 397-408, doi:10.1111/jmi.12579 (2017).
5. Kolossov, V. L. *et al.* Airyscan super-resolution microscopy of mitochondrial morphology and dynamics in living tumor cells. *Microsc Res Tech*, doi:10.1002/jemt.22968 (2017).
6. Singh, R. *et al.* Real rock-microfluidic flow cell: A test bed for real-time in situ analysis of flow, transport, and reaction in a subsurface reactive transport environment. *J Contam Hydrol* **204**, 28-39, doi:10.1016/j.jconhyd.2017.08.001 (2017).
7. Urban, M. A., Barclay, R. S., Sivaguru, M. & Punyasena, S. W. Cuticle and subsurface ornamentation of intact plant leaf epidermis under confocal and superresolution microscopy. *Microsc Res Tech*, doi:10.1002/jemt.22667 (2016).
8. Huff, J. The Airyscan detector from ZEISS: confocal imaging with improved signal-to- noise ratio and super-resolution. *Nat Methods* **12** (2015).
9. Schneider, C. A., Rasband, W. S. & Eliceiri, K. W. NIH Image to ImageJ: 25 years of image analysis. *Nat Methods* **9**, 671-675 (2012).
10. Primiano, A. *et al.* FT-IR analysis of urinary stones: a helpful tool for clinician comparison with the chemical spot test. *Dis Markers* **2014**, 176165, doi:10.1155/2014/176165 (2014).
11. Cromey, D. W. Avoiding twisted pixels: ethical guidelines for the appropriate use and manipulation of scientific digital images. *Sci Eng Ethics* **16**, 639-667, doi:10.1007/s11948-010-9201-y (2010).

**III. Supplementary Figures**

**
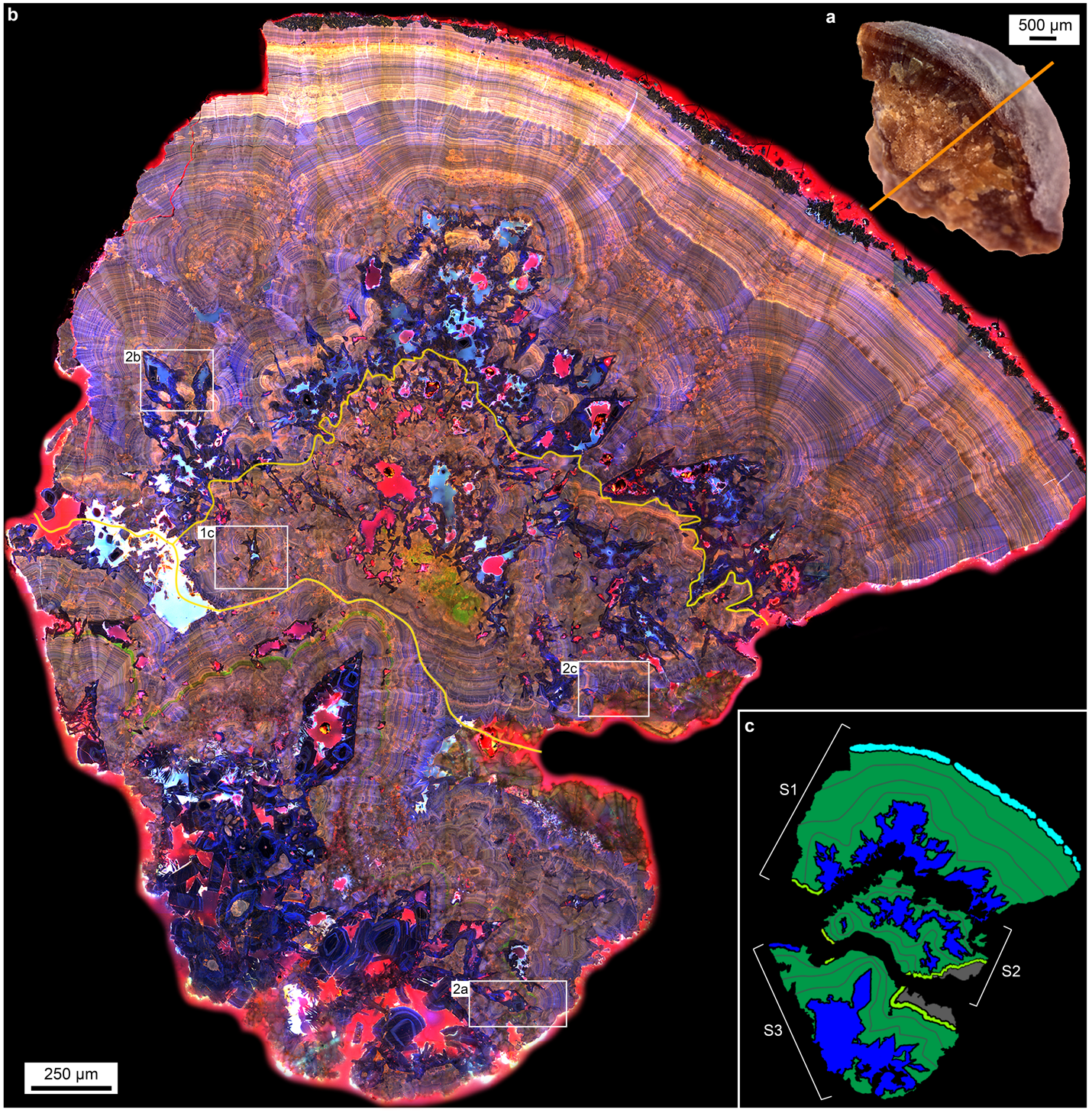
Supplementary Figure 1 | Crystalline architecture of the MP2 calcium oxalate (CaOx) kidney stone. a**, Bright field (BF) image of the MP2 stone showing the position and orientation of thin section preparation (orange line). The brightness and contrast of the image is adjusted to clearly document the whole stone morphology prior to sectioning. The raw image with and without adjustments is presented in Supplementary Figure 19. **b**, Tiled confocal auto-fluorescence (CAF) image composed of merged pseudo-colored red, green and blue (RGB) channels. Red and light blue auto-fluorescence (AF), which is emitted from the embedding epoxy, has been cropped and replaced by a black background. Boxes indicate the location of enlargements shown in Figs. 1, 2. Image (**b**) is displayed with best-fit intensity profiles after a gamma correction of 0.70 (raw images with and without adjustments are presented in Supplementary Figure 20). **c**, Detailed tracing of the MP2 stone in (**b**) illustrates that it is actually composed of an interlocked complex of three individual stone fragments (each fragment margin is traced yellow in (**b**, see also Supplementary Video 1). Tracing legend includes: dark blue = CODFF aggregates replaced by COMR crystals; green = COMC; yellow = COMCE; gray = UACE; cyan = COMR and CODR.


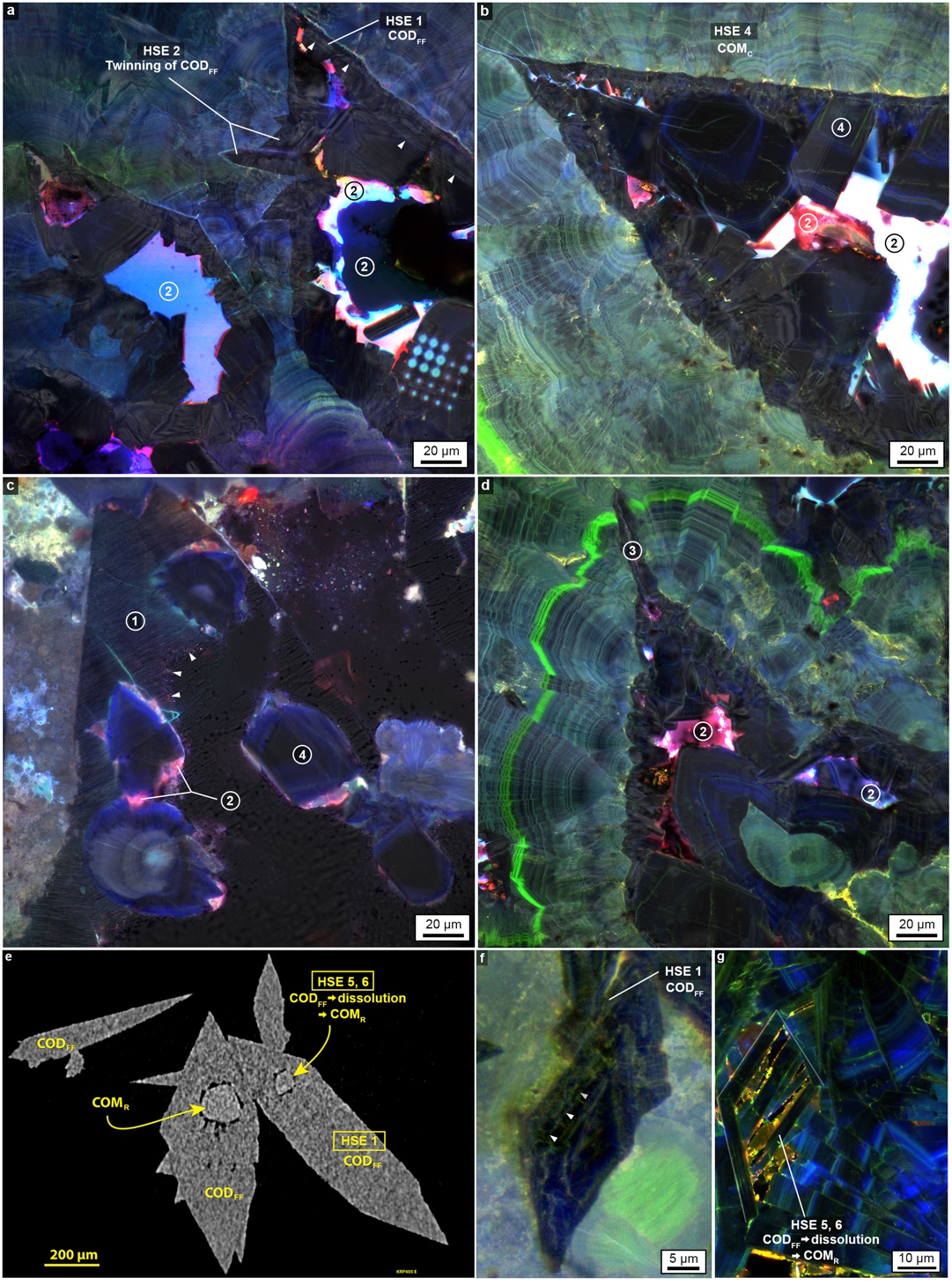


**Supplementary Figure 2 | Dissolution recorded by four types (1-4) of fabrics that cross-cut the crystalline architecture representing all HSE events in the MP-series kidney stones. a-d,** BF image overlay on SRAF image of merged RGB channels demonstrate dissolution recorded by four types of fabrics, which are referenced as numbers (1) to (4). (**a**) Euhedral CODFF growth extensions (HSE 2) from the outermost surfaces of larger CODFF (HSE 1), reflecting twinned CODFF. CODFF then dissolves, leaving remnants (white arrowheads) and creating moldic porosities (2). (**b**) A dense nano-layered COMC (HSE 4) encrusting CODFF. CODFF then dissolves, leaving moldic porosity (2) filled with COMR (4). (**c**) Irregular void spaces lined with remnants of partially dissolved CODFF (1). White arrowheads denote dissolved edge of CODFF. Some porosity is filled with COMR (2). (**d**) Dissolution creates canyon-like void spaces that cross-cut COMC nano-layering (3). **e,** Micro-CT image cross- sectional slice of COD crystals in a CaOx kidney stone collected during an ureteroscopic procedure at Indiana University Purdue University Indianapolis Hospital. This Micro-CT image was collected on a Skyscan 1172 System using 60 kVp source mounted with a 0.6 mm A1 filter, undergoing rotation steps of 0.7 degree. Voxel size of final reconstructed image was 2.9 µm. The interior of COD has been dissolved and replaced with COMR, reflecting similar fabrics shown in (**c**). **f,** SRAF image of merged RGB channels show CODFF partially dissolving and leaving original crystal remnants (white arrowheads) around the margins. **g,** SRAF image of merged RGB channels show CODFF dissolving completely. The white outline denotes the original CODFF that has been replaced by COMR lathes. In images (**a**) through (**d**), brightness and contrast are adjusted and a BF overlay is applied to highlight the crystalline architecture, as demonstrated in Supplementary Figure 21. Image (**f**) is displayed with a best-fit intensity profile after a gamma correction of 0.45. Image (**g**) is displayed with linear brightness and contrast adjustments as presented in Supplementary Figure 22.


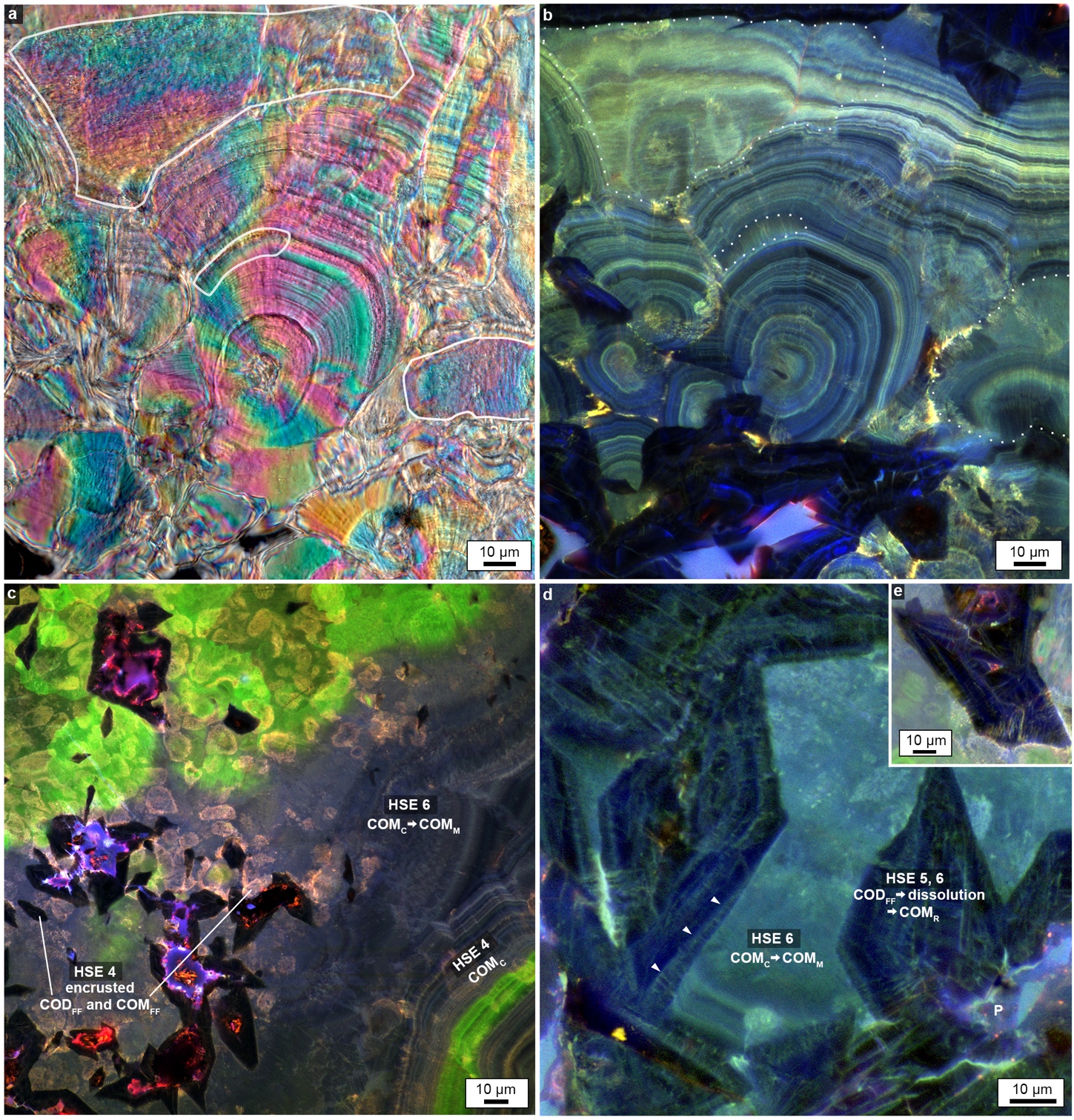


**Supplementary Figure 3 | Evidence of dissolution and crystallization at both the Ångstrom-scale (mimetic replacement) and micron-scale representing HSE 4-6 in the MP-series kidney stones. a, b,** Solid white line traces in a CPOL image (**a**), as well as dotted lines in an SRAF image of merged RGB channels (**b**) outline regions of COMM. This represents Ångstrom-scale dissolution and subsequent crystallization of COMC. In some locations, “smudge-looking” layers of COMC can be seen, as it incrementally begins to dissolve and crystallize into COMM. **c,** SRAF image of merged RGB channels shows a generation of CODFF and COMFF (HSE 4) encrusted within a large area of COMM (HSE 6). **d-e** Higher magnification image shows encrusted CODFF are completely dissolved at the scale of tens of microns, forming porosity (P) that is then filled with lathes of COMR (HSE 5, 6). Additionally, CODFF dissolves only partially, leaving CODFF remnants (white arrowheads). Image (**a**) is displayed with best-fit intensity profiles after a gamma correction of 0.45, as described in Supplementary Figure 18. Images (**b**) and (**c**) are displayed with linear brightness and contrast adjustments, as presented in Supplementary Figure 22. Images (**d**) and (**e**) are displayed with best-fit intensity profiles after a gamma correction of 0.45.


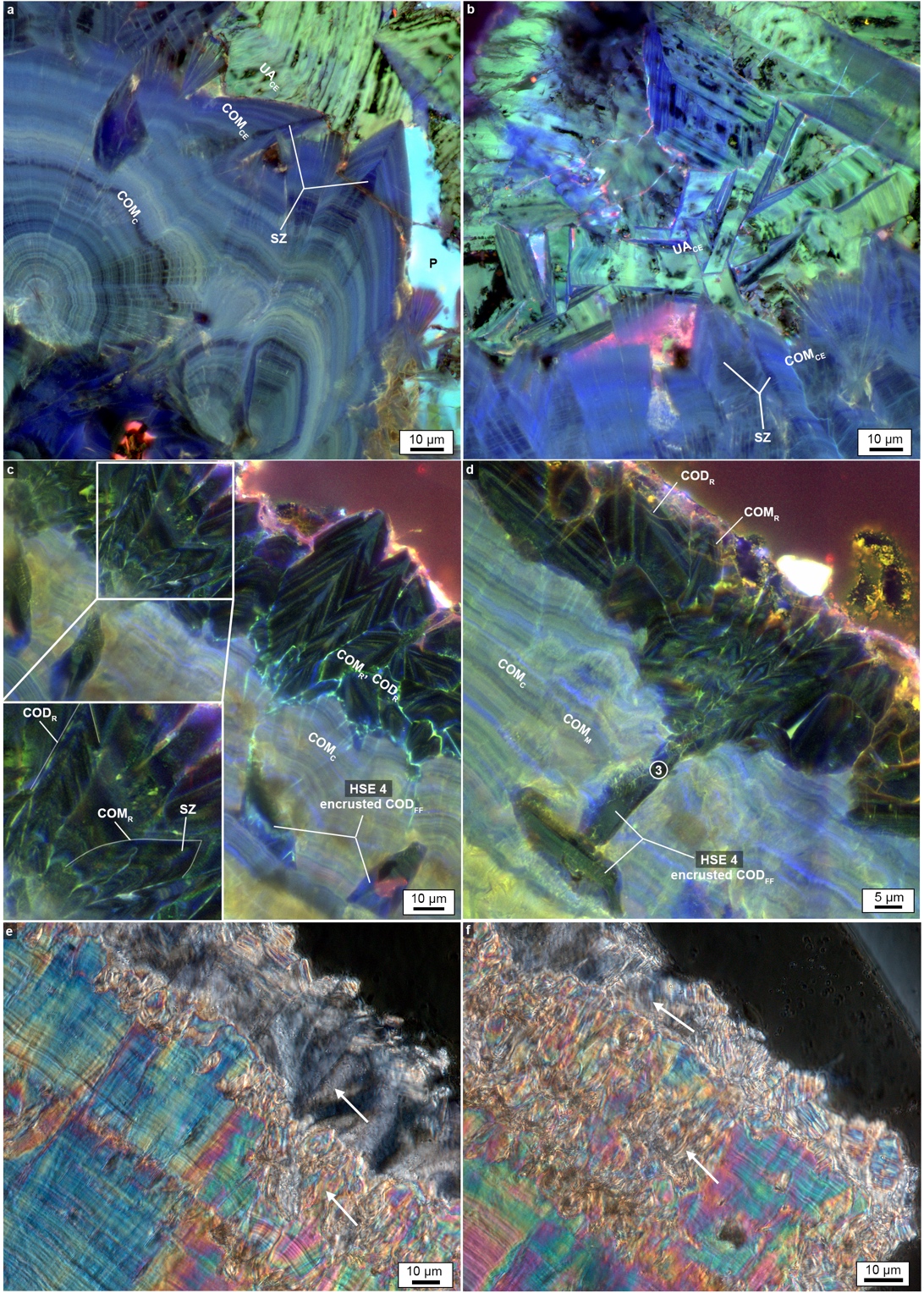


**Supplementary Figure 4 | Late stage dissolution and crystallization on the outermost margins of the MP2 CaOx kidney stone. a, b,** SRAF images of merged RGB channels show late stage crystal growths of CaOx and uric acid on the outermost margins of the kidney stone. On the surfaces of COMC, COM crystals grow as cements (COMCE). COMCE exhibits the characteristic rounded crystal faces and sector zoning (SZ) of COM crystals. As the final stage, uric acid cement crystals (UACE) grow on top of the COMCE. **c, d,** On other outer surfaces of the kidney stone, COMC dissolves and is recrystallized as COD and COM (CODR and COMR). Rounded crystal faces and SZ differentiates COMR from CODR, which has sharp geometric faces and no SZ (white outlines in inset). Images (**c**) and (**d**) also show late stage CODFF landing on outer COMC cortex layers (HSE 4). **d**, ensuing dissolution of both CODR and COMR cuts down into COMC, resulting in canyon-like void space fabrics (3) presented in Supplementary Figure 2d**.** In addition, image (**d**) exhibits COMM mimetic replacement of COMC. **e, f,** CPOL images of the outermost margins of the kidney stone in locations similar to (**c**) and (**d**). Arrows show significantly larger crystallites, indicative of both COMFF and CODFF landing on and being encrusted by COMC (HSE 4). In addition, partial dissolution and replacement of COMC by CODR and COMR is documented by a distinct difference in birefringence: COMC exhibits higher birefringence (range of pastel colors) while CODR and COMR exhibit lower birefringence (gray to black colors). Images (**a**-**d**) are displayed with brightness and contrast adjustments as presented in Supplementary Figure 22 after a gamma correction of 0.45. Images (**e**) and (**f**) are displayed with best-fit intensity profiles after a gamma correction of 0.45, as described in Supplementary Figure 18.


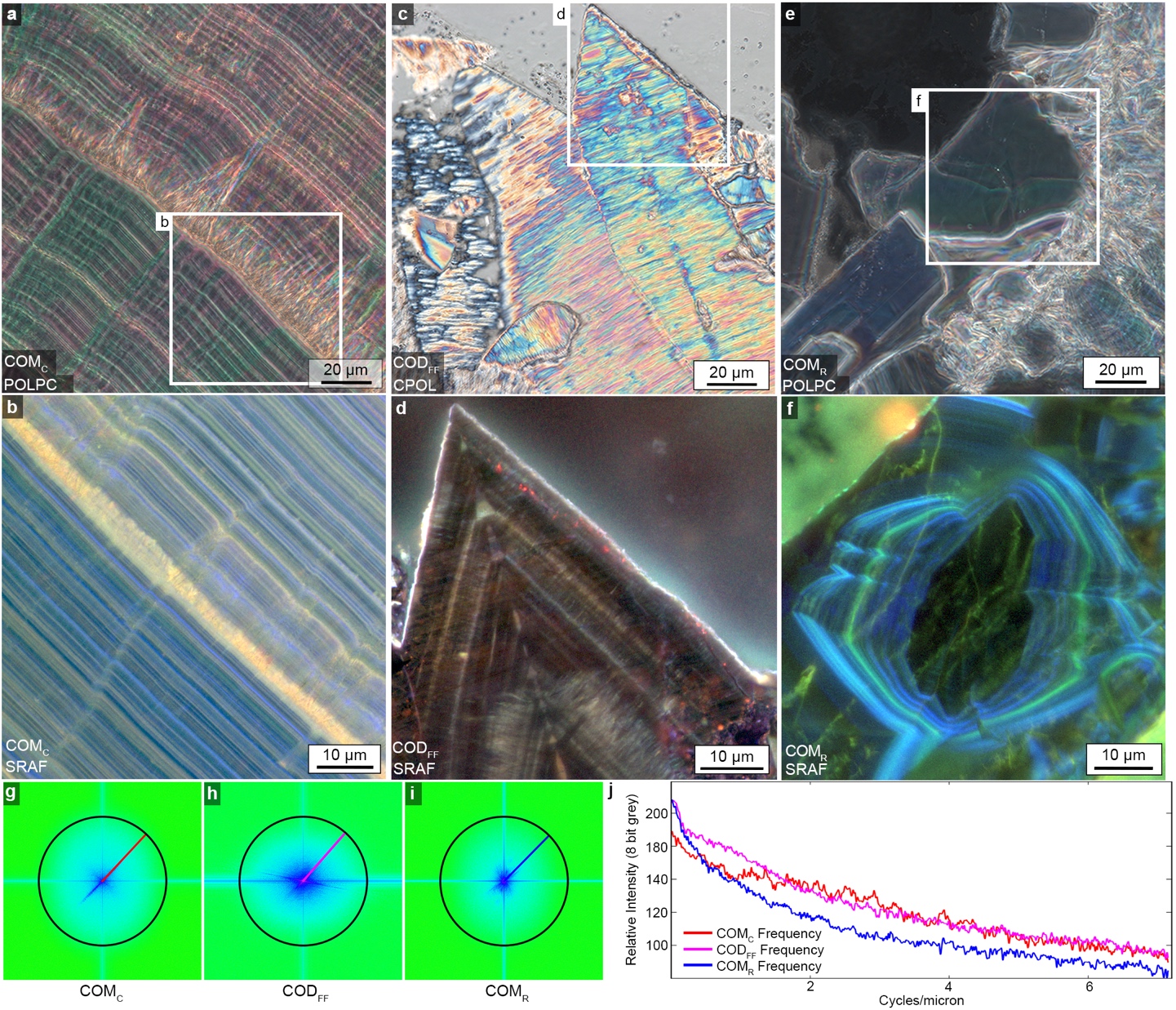


**Supplementary Figure 5 | Comparison of nano-layering in COMC, CODFF and COMR crystals of the MP2 CaOx kidney stone. a**, POLPC image of COMC crystals. **b**, SRAF image of merged pseudo-colored RGB channels showing COMC nano-layers (enlargement of box in **a**). **c**, CPOL image of CODFF crystals. **d**, SRAF image of merged pseudo-colored RGB channels showing CODFF internal concentric nano-layering crystal zoning (enlargement of box in **c**). **e**, POLPC image of COMR crystal. **f**, SRAF image of merged pseudo-colored RGB channels showing COMR internal concentric nano-layer crystal zoning (enlargement of box in **e**). **g**-**i,** FFT images of **b**, **d** and **f**, respectively. Green represents highest frequencies, cyan represents the full extent of the frequencies retrieved by the system and black circles in each FFT represent a resolution of 140 nm. **j,** Graph of crystal layering frequencies plotted as intensities against cycles/micron. Each curve represents its corresponding color transect line shown in the FFT images (**g**-**i**). The x-axis ends at ~7.14 cycles/micron, which represents ~140 nm resolution. Images (a-f) are displayed with best-fit intensity profiles after a gamma correction of 0.45 (as described in Supplementary Figure 18). No adjustments have been made to figures **g**-**j.** Point-by-point raw data in Excel format for (**j**) is available to download.


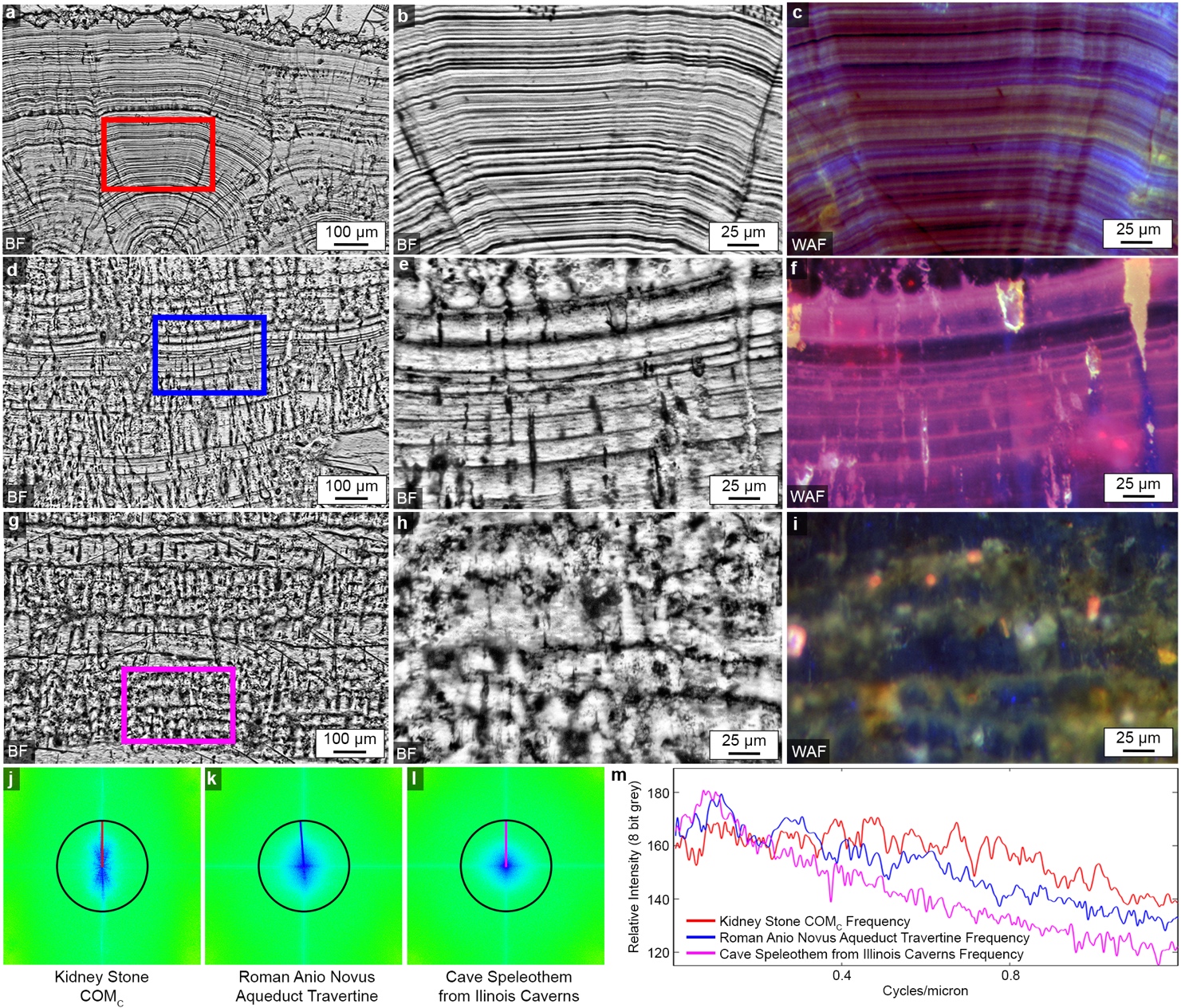


**Supplementary Figure 6 | Comparison of COMC nano-layering in the MP2 CaOx kidney stone with layering observed in cave and aqueduct deposits. a,** BF image of COMC nano-layering in the MP2 kidney stone. **b**, Enlargement of box in (**a**) **c**, Wide- field auto-fluorescence (WAF) image of merged pseudo-colored RGB channels at the same location shown in (**b**). **d**, BF image of layering in Roman aqueduct travertine deposits. **e**, Enlargement of box in (**d)**. **f**, WAF image of merged pseudo-colored RGB channels at the same location shown in (**e**). **g**, BF image of layering in cave speleothem deposits. **h**, Enlargement of box in (**g**). **i**, WAF image of merged pseudo-colored RGB channels at the same location shown in (**h**). **j**-**l,** FFT images of **c**, **f** and **i**, respectively. Green represents the highest frequencies, cyan represents the full spectrum of frequencies collected by the microscope system, and black circles in each FFT represent a resolution of 1.2 µm. **j,** Graph of crystal layering frequencies plotted as intensities against cycles/micron. Each curve represents its corresponding color transect line shown in the FFT images (**j**-**l**). The x-axis at 0.82 cycles/micron represents ~1.2 m resolution. Images **a**-**h** are processed with CLAHE filter in the program Image J. Images in **c**, **f** and **i** are displayed in best-fit mode. Point-by-point raw data in Excel format for (**m**) is available to download.


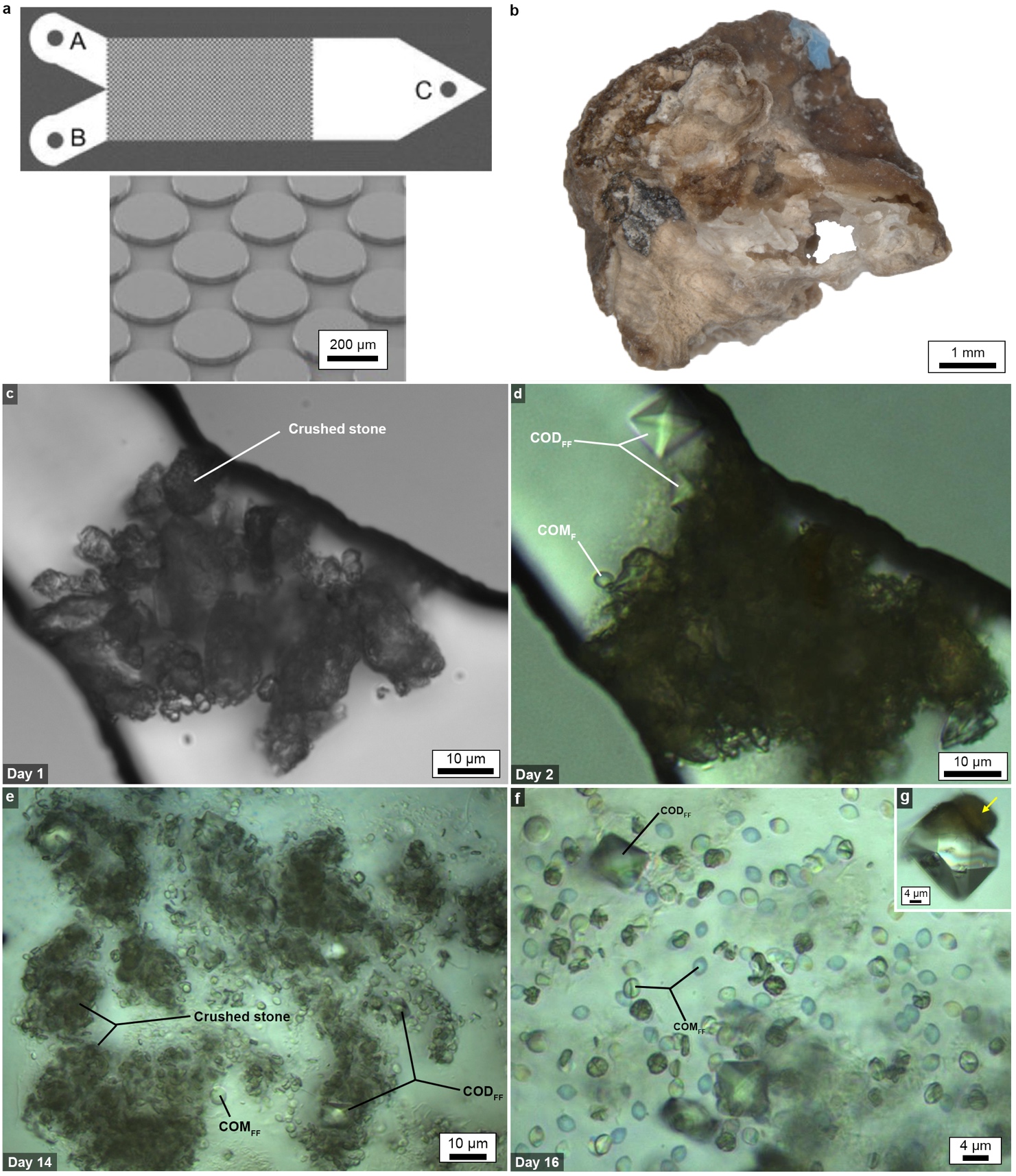


**Supplementary Figure 7 | *In vitro* polymorphic CaOx crystallization within a microfluidic device that recreates HSE 1. a,** Model of microfluidic device with enlargement of pore area (details in Methods). **b**, CaOx stone fragment that was crushed, sieved and injected into the microfluidic device. **c**, Black and white BF image of crushed stone particles trapped in a silicon pore throat at day 1 of urine flow through the micromodel. **d**, BF image of CODFF and COMFF growing on the surface of crushed stone particles on day 2. **e**, BF image showing a location near the outlet of the device where CODFF and COMFF grow amongst crushed stone particles at day 14. **f**, A higher magnification BF image showing CODFF and COMFF growing amongst crushed stone particles at day 16. **g**, BF image exhibits a single CODFF, distinguished by the characteristic bi-pyramidal shape, growing on the surface of a crushed stone particle (yellow arrow) in the microfluidic device (see also additional data in Supplementary Figure 14).


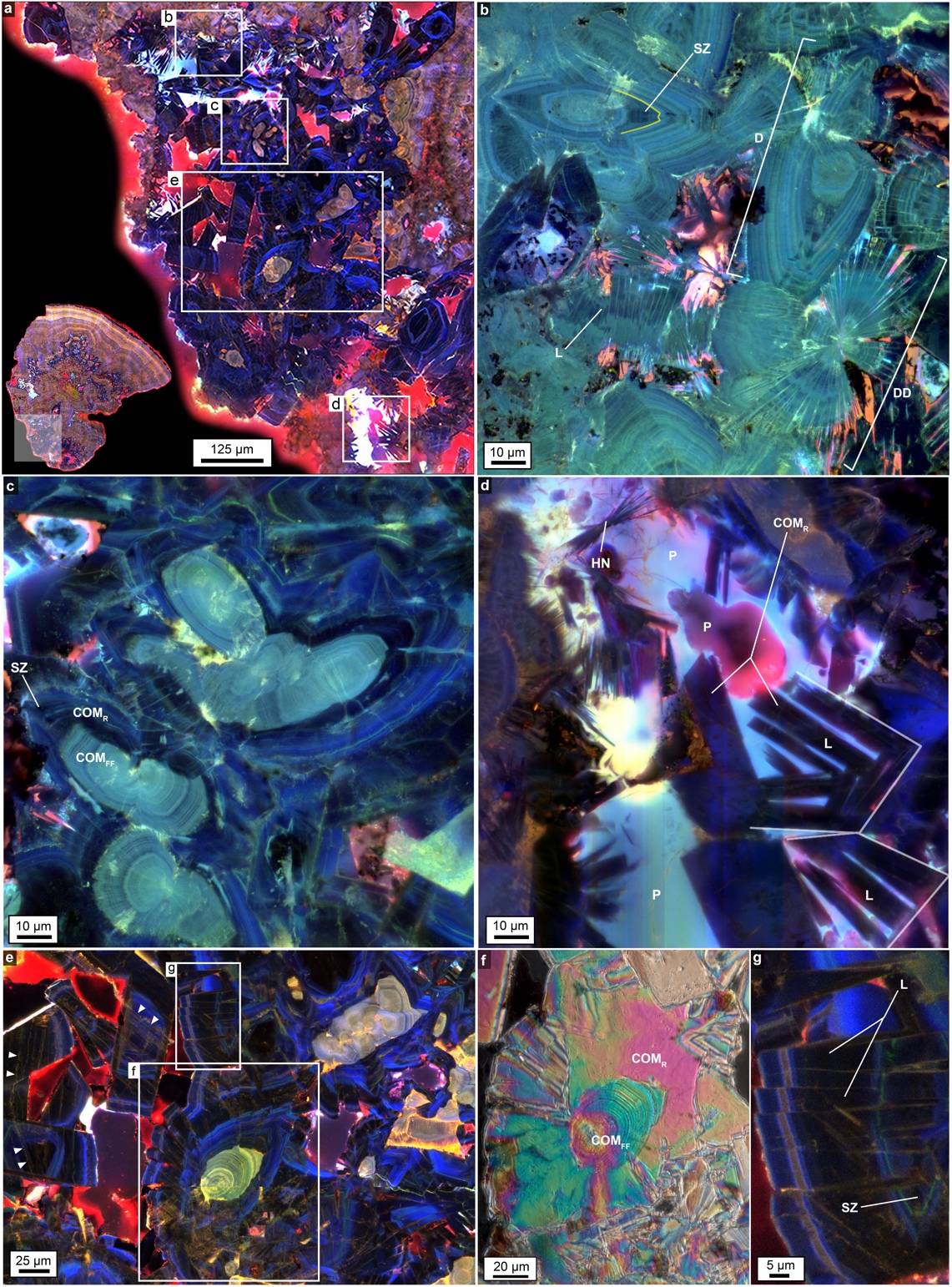


**Supplementary Figure 8 | Polymorphic CaOx crystals comprising the MP2 CaOx kidney stone. a**, Tiled CAF image of merged pseudo-colored RGB channels of stone fragment 3 (S3), shown as an enlargement of the highlighted inset at bottom left corner. **b**-**d**, Enlargement of boxes in (**a**) shown in (**b**, **c**, and **d**) as SRAF images of merged pseudo-colored RGB channels, which demonstrate: (**b**) Polymorphs of COMFF, including lathes (L), dumbbells (D), double dumbbells (DD). Additionally, some crystals are identified as a polymorph of COM by their rounded crystal faces (yellow outline) and sector zoning (SZ). (**c**) Outer surfaces of dumbbell polymorphs of COMFF dissolve and are replaced by COMR with rounded crystal faces and SZ. (**d**) COM polymorphs, including “haystack needles” (HN) and lathes (L) grow in moldic porosity (P) left behind by completely dissolved CODFF. The white outlines denote the original CODFF that has been replaced by COMR lathes. **e**, Enlargement of box in (**a**) under CAF merged pseudo-colored RGB channels shows polymorphic CaOx crystals. **f,** Enlargement of a box in (**e**) under CPOL shows dissolution of COMFF followed by COMR crystallization. **g**, Enlargement of a box in (**e**) under CAF showing COMR in the form of lathes (L). Crystals also exhibit rounded crystal faces with sector zoning (SZ) characteristic of COM. No adjustments were made in images (**a**) and (**e**). Images (**b**) and (**c**) are displayed with brightness and contrast adjustments (presented in Supplementary Figure 22) after a gamma correction of 0.45. Images (**d**) and (**f**) are displayed with best-fit intensity profiles after a gamma correction of 0.45 as presented in Supplementary Figure 18.


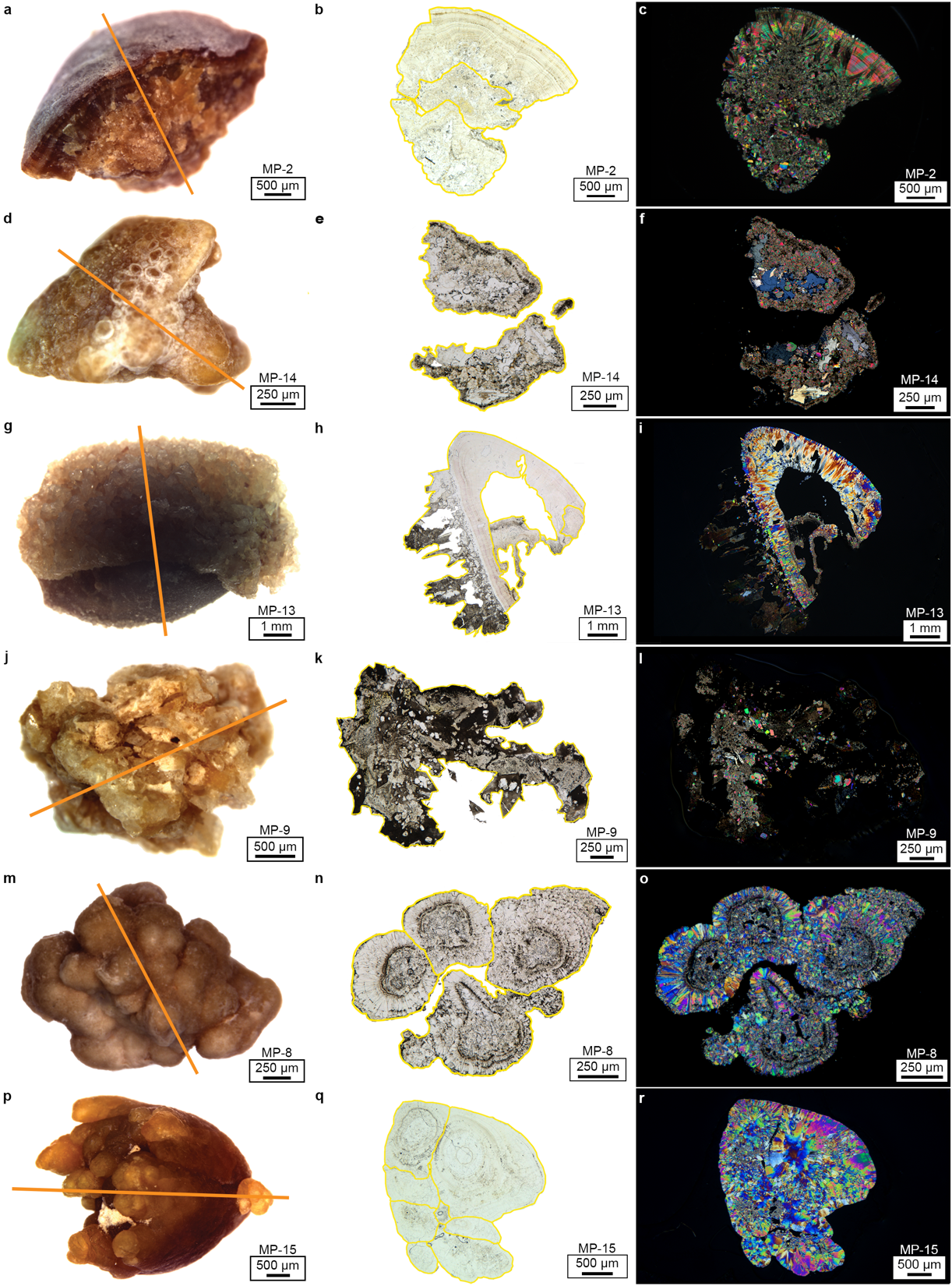


**Supplementary Figure 9 | Six Mayo patient MP-series kidney stones showing whole stone morphology and crystalline architecture in thin section under BF and POL microscopy. a**, **d**, **g**, **j**, **m**, **p**, BF images of whole stones, which are labeled as part of the MP-series Mayo Patient sample catalogue. Orange line indicates orientation of the line of section along which each stone thin section was prepared. **b**, **e**, **h**, **k**, **n**, **q**, BF images of each whole stone thin section illustrates that each sample is composed of multiple stone fragments (yellow lines are tracings that indicate multiple stone fragments merged to form a single stone complex. **c**, **f**, **i**, **l**, **o**, **r**, Polarization images of stone thin sections demonstrate the multitude of orientations of crystals within the stone. Infra-red spectral analysis of each stone has identified the following mineral compositions: (**a-c**), MP2 (100% COM); (**d-f**), MP14 (100% COM); (**g-i**), MP13 (100% COD); (**j-l**), MP9 (70% COD, 20% COM, 10% apatite); (**m-o**), MP8 (60% COD, 20% COM, 20% apatite); (**p-r**), MP15 (60% COD, 30% apatite 10% COM). All images in this panel are displayed with linear brightness and contrast adjustments as described in Supplementary Figure 19.


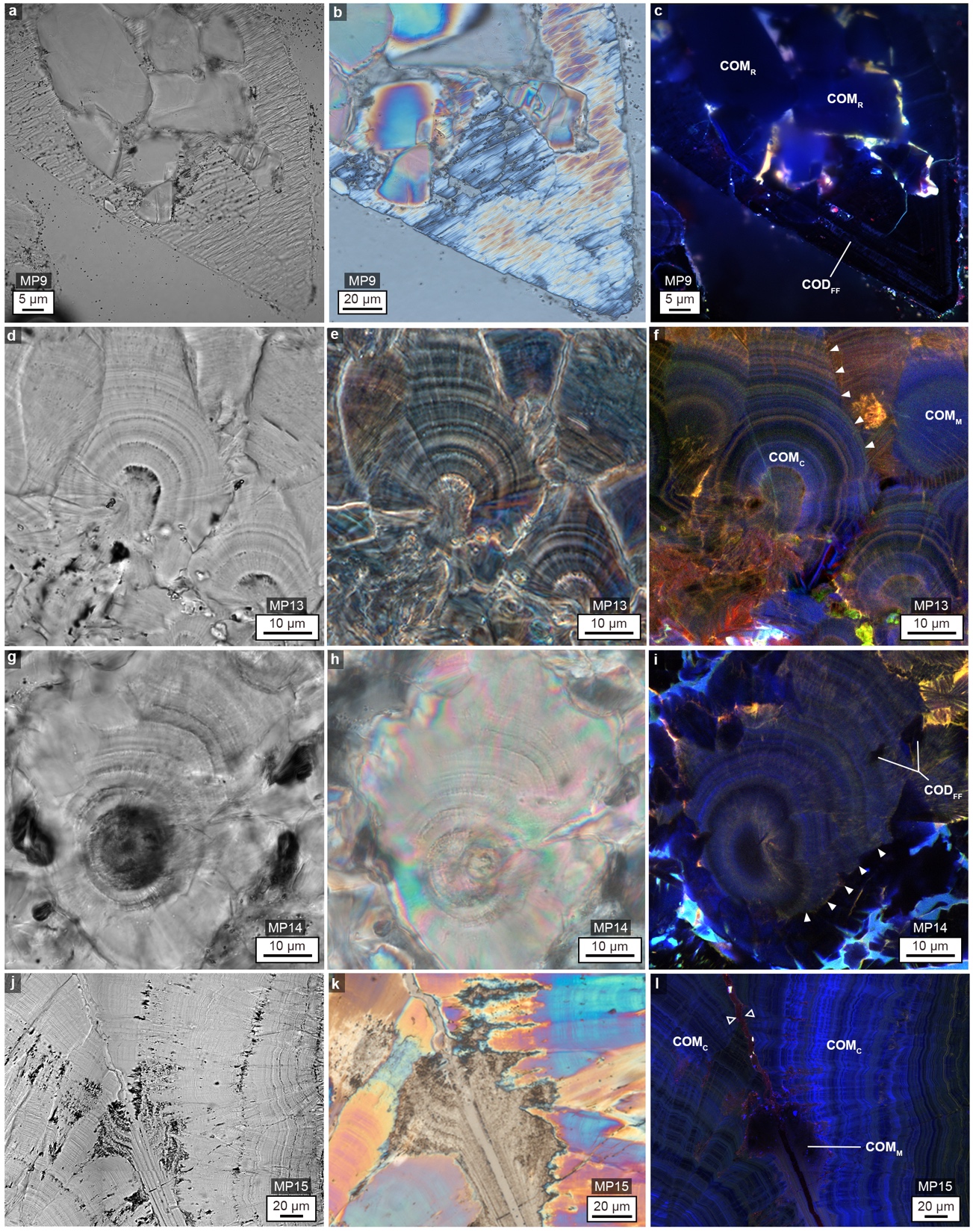


**Supplementary Figure 10 | Extensive and repeated dissolution, crystallization and ultra-high frequency layering in MP-series kidney stones representing all HSE events.** BF, POL and SRAF images of merged RGB channels are in the left (red), middle (green) and right (blue) columns, respectively. See Supplementary Figure 9 for whole-stone morphology, IR analysis, and thin section images. **a-c,** Thin section of MP9 shows CODFF dissolved and replaced by COMR. **d-f**, Thin section of MP13 shows nano-layering of COMC, truncated layering from dissolution (arrowheads), and mimetic replacement of COMC by COMM. **g-i**, Thin section of MP14 shows nano-layering of COMC with CODFF and COMFF landing and encrusted on the COMC. Arrowheads depict edges of extensive dissolution. **j-l**, Thin section of MP15 showing the margin of two stone fragments where COMC nano- layering from each stone fragment came into contact with each other (open arrowheads). At this location, COMC is mimetically replaced by COMM. BF (**a**, **d**, **g**, and **j**) and SRAF (**c**, **f**, **i**, **l**) images are displayed with brightness and contrast adjustments as presented in Supplementary Figure 22. POL images (**b**, **e**, **h**, **k**) are displayed with best-fit intensity profiles after a gamma correction of 0.45, as described in Supplementary Figure 18.

**
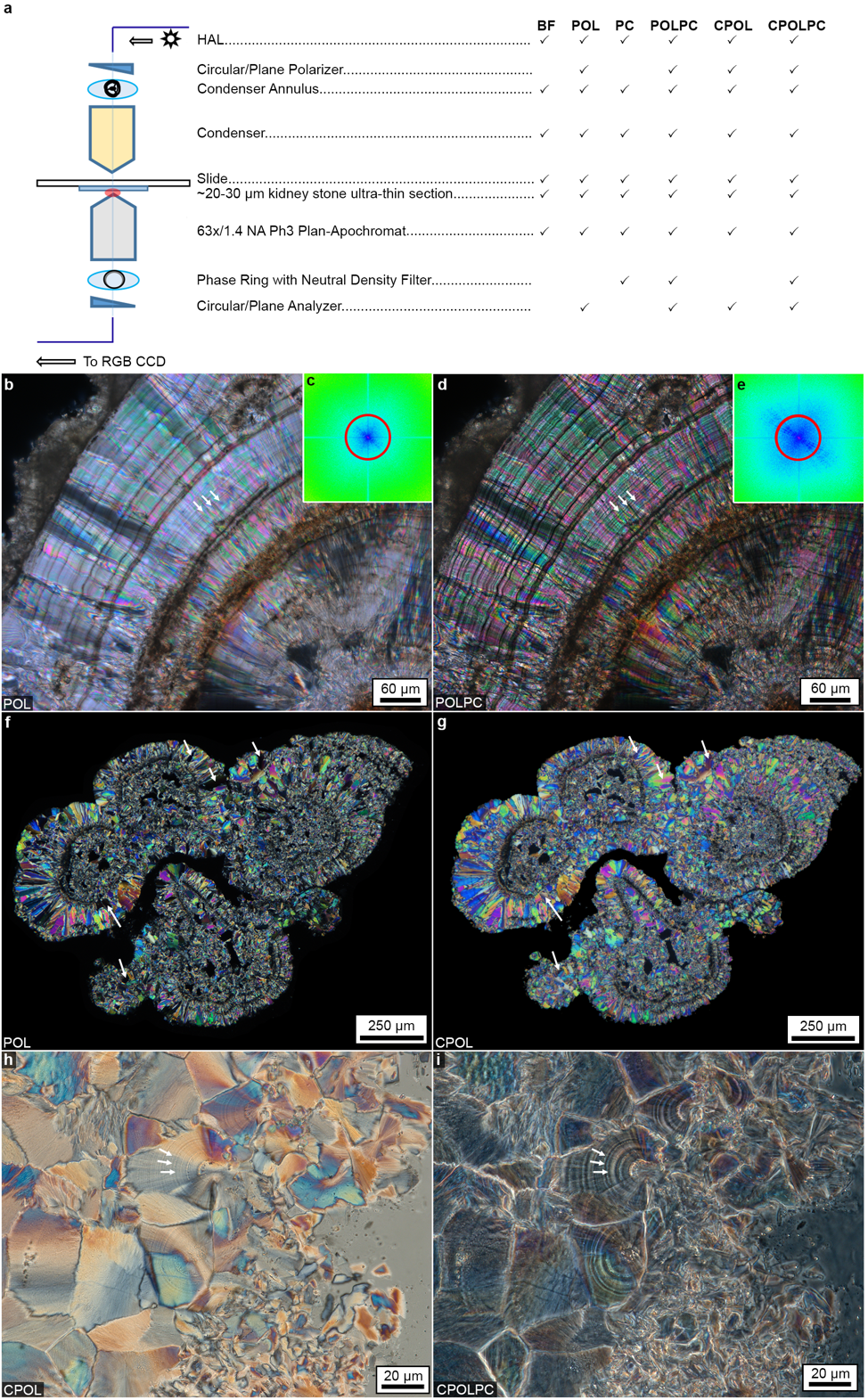
**

**Supplementary Figure 11 | Optical techniques applied to MP-series kidney stones in the present study. a,** Schematic of transmission light-path for bright field (BF), polarization (POL), phase contrast (PC), polarization phase contrast (POLPC), circularized polarization (CPOL), and circularized polarization with phase contrast (CPOLPC). **b**, Kidney stone image under crossed Nicol prisms (POL). **c,** Fast Fourier transformation (FFT) of image (**b**). The maximum extent of frequencies retrieved by the POL technique is indicated by the red circle in (**c**). **d**, POLPC image of the kidney stone at the same location as (**b**). **e,** FFT of image (**d**) with the red circle from (**c**) superimposed. The blue pixels in (**e**) fills the red circle more densely and extends past the red circle. This indicates that the POLPC image has higher resolution than the POL image (arrows in (**b**) and (**d**), which compare the resolution and contrast in layering between the two techniques). **f, g,** POL image of a kidney stone under crossed Nicol prisms (**f**) in comparison with CPOL image of the same stone (**g**). The CPOL technique illuminates all crystals irrespective of their axis of orientation (arrows indicate the crystals that are black in the POL image, but birefringent and visible in the CPOL image). **h**, **i,** CPOL image of a kidney stone (**h**) and its corresponding CPOLPC image (**i**). The CPOLPC technique illuminates all concentric fine layering with better resolution and contrast compared to CPOL (arrows). Images (**b**, **d**, **f** and **g**) are presented with best-fit intensity profiles. Images (**h**) and (**i**) are displayed with min-max intensity profiles after a gamma correction of 0.45. Other optical techniques such as CAF, SRAF, TPAF and FLIM techniques used in this study are described in detail in Methods.


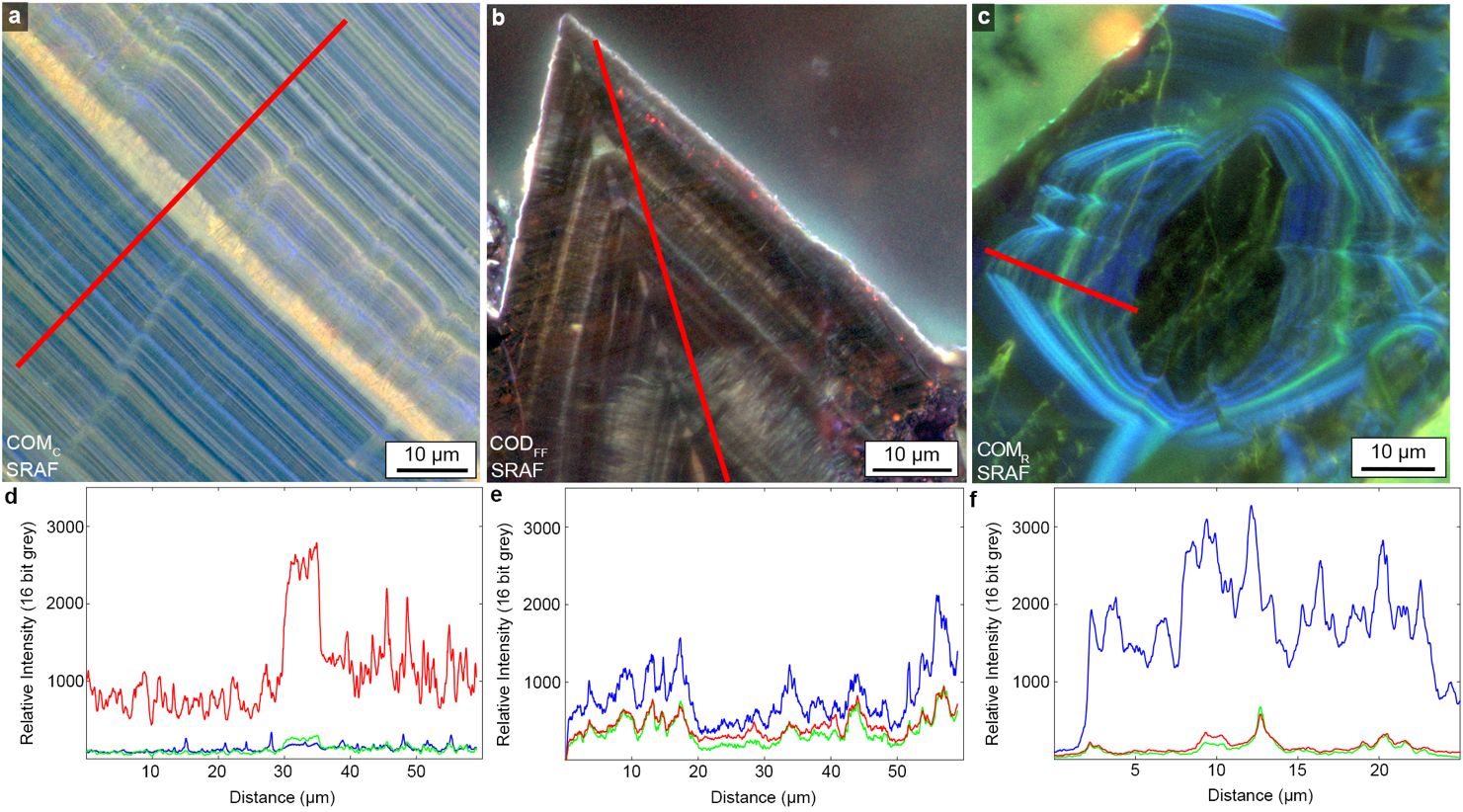


**Supplementary Figure 12 | Comparison of deposited organic matter AF in nano-layers among COMC, CODFF and COMR within the MP-series kidney stones. a-c**, SRAF images of merged pseudo-colored RGB channels showing nano-layers of COMC (**a**), CODFF (**b**) and COMR (**c**). **d**-**f**, Intensity profiles of line transect shown in each SRAF image in three RGB channels (red line overlay) show AF channel peaks and troughs exhibited in COMC (**d**) , CODFF (**e**), and COMR (**f**). Peaks represent organic matter-rich layers and troughs represent crystal-rich layers (line colors in the graph correspond to their RGB channels). Note the intensity profiles of CODFF and COMR, where the pattern of red and green channel intensities follow each other closely and the blue channel exhibits the highest intensities in both crystals. These patterns are not exhibited in COMC, which shows the highest intensities in the red channel. Images in (**a**-**c**) are displayed with best-fit intensity profiles after a gamma correction of 0.45, as described in Supplementary Figure 8. Point-by-point raw data in Excel format for **d, e and f** are available to download.


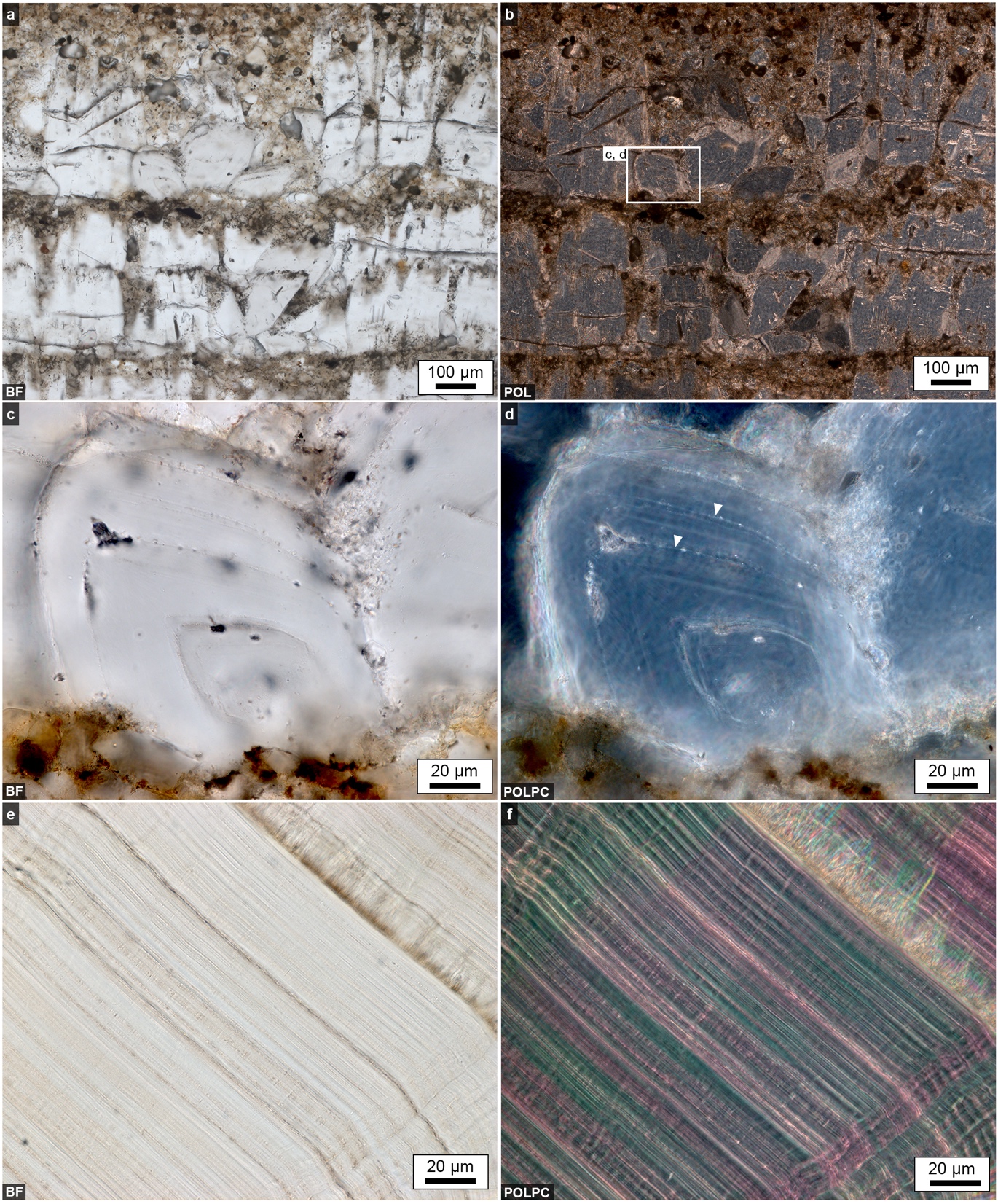


**Supplementary Figure 13 | Evidence for the necessity of using a lower magnification optical system for comparing**

**layering in the MP-series kidney stones and other natural environments. a,** BF image of cave limestone layering in Illinois Caverns. **b,** Same location as (**a**) under off-axis polarized light. **c,** High magnification (63x/1.4 NA Plan Apochromat Ph3 Oil immersion) BF image of box in (**b**). **d**, Same location as (**c**) under POLPC. **e,** High magnification BF image showing COMC nano-layering in MP2 kidney stone. **f**, Same location in (**e**) under POLPC. These images indicate that the layering observed in natural cave environments is composed of substantially larger crystallites and thus thicker layers, where even a single layer is too large to be imaged under the high magnification. However, note that concentric zonations within these larger crystals can be detected at this magnification (arrows in **d**), which are much closer in thickness and frequency to those observed in the MP-series kidney stones. Images (**a**-**d**) are displayed with a gamma correction of 0.45. Images (**e**) and (**f**) are displayed with min-max intensity profiles after a gamma correction of 0.45.


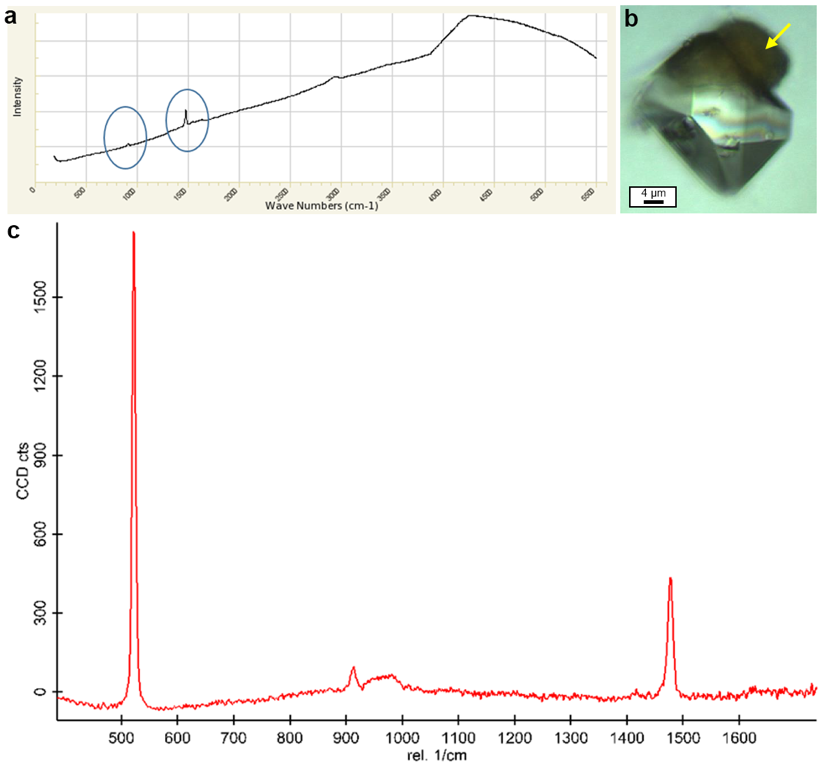


**Supplementary Figure 14 | Raman spectra confirming the mineralogical composition of COD grown *in vitro* in a microfluidic device. a**, Raman spectra for COD from the RRUFF online database showing distinct COD Raman peaks at wave- lengths of 912 and 1477. **b**, BF image shows a single CODFF crystal, distinguished by the characteristic bi-pyramidal shape, growing on the surface of a crushed CaOx stone particle (yellow arrow) in the microfluidic device (see also additional data in Supplementary Figure 7). **c,** Actual COD spectra obtained from COD crystal in (**b)** showing matching Raman spectra as in the RRUFF online database, with characteristic COD peaks at wave numbers 912 and 1477. The large peak at 532 identifies and matches the Raman spectra for silicon.


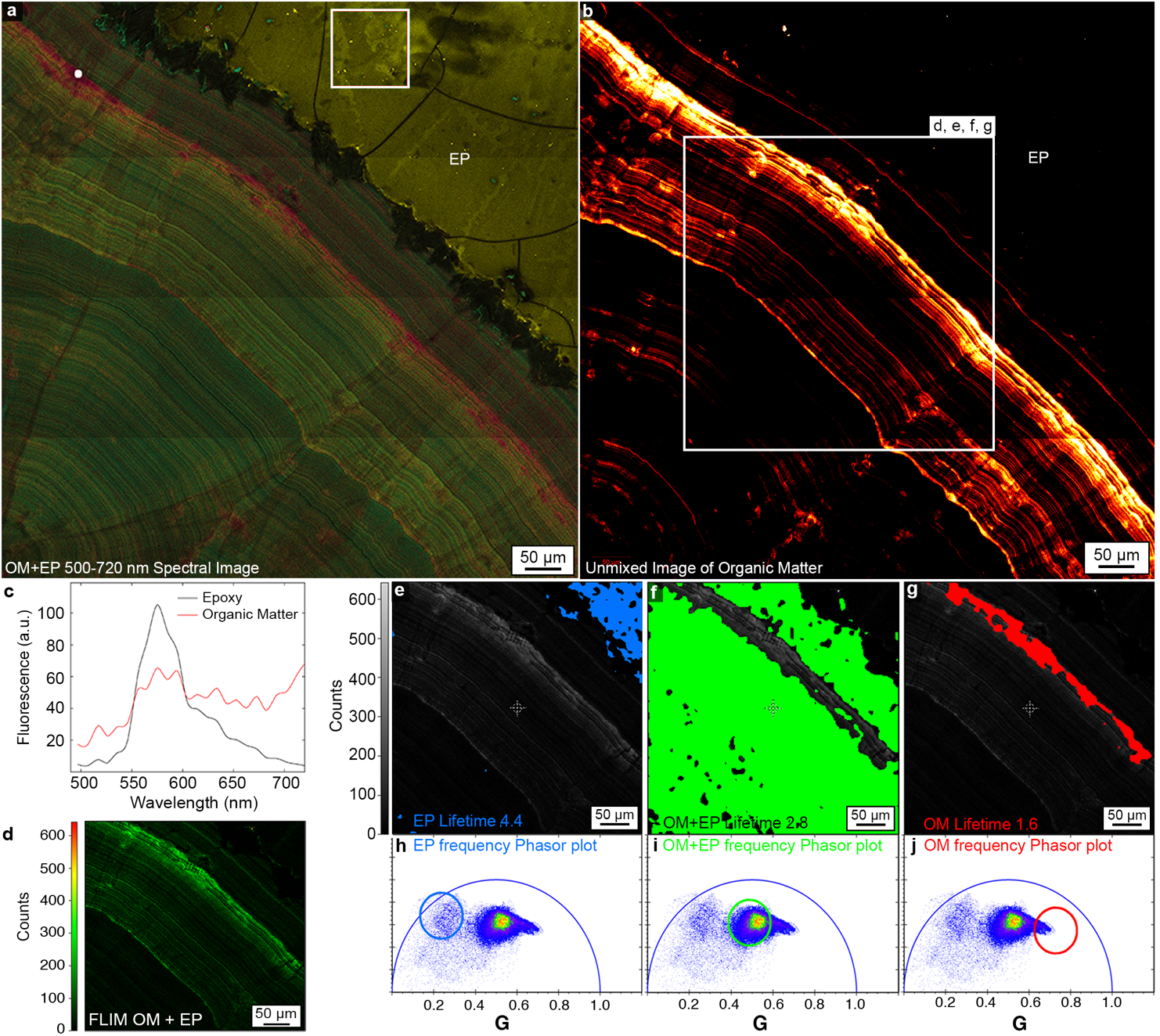


**Supplementary Figure 15 | Characterization of the distribution and composition of COMC organic matter-rich nano- layers using two-photon spectral and fluorescence lifetime imaging (FLIM) in the MP2 CaOx kidney stone. a**, A total of 16 tiled images from 4 x 4 individual TPAF spectral images using 20 different detectors at 10 nm spectral resolution and colored according to the emission spectra of both epoxy (EP) and organic matter (OM). Using a spectral un-mixing algorithm, a small area (white spot) represents analysis of a dense organic matter-rich COMC layer. The epoxy emission signal, determined from a much larger area (white box) was subtracted from analyses of the organic matter-rich areas. The entire image was subsequently processed based on this characterization. **b**, Emissions from the pure organic matter-rich nano-layers, visualized by the approach described in (**a**). **c**, A spectral graph of the image locations shown in (**a**). **d**, FLIM image of the region shown in (**b**). **e**, Segmentation of FLIM image showing the pure epoxy signal (blue) and the location of its corresponding lifetime frequencies (blue circle in the corresponding phasor plot, **h**). **f**, Segmentation of FLIM image showing the mixed epoxy and organic matter signal (green) and the location of its corresponding lifetime frequencies (green circle in the corresponding phasor plot, **i**). **g**, Segmentation of FLIM image showing the pure organic matter signal (red) and the location of its corresponding lifetime frequencies (red circle in the corresponding phasor plot, **j**). The brightness and contrast of the individual images (**a** and **b**) are adjusted and presented in either min/max or best-fit mode for optimal viewing. No adjustments were made on images **c**-**j**.


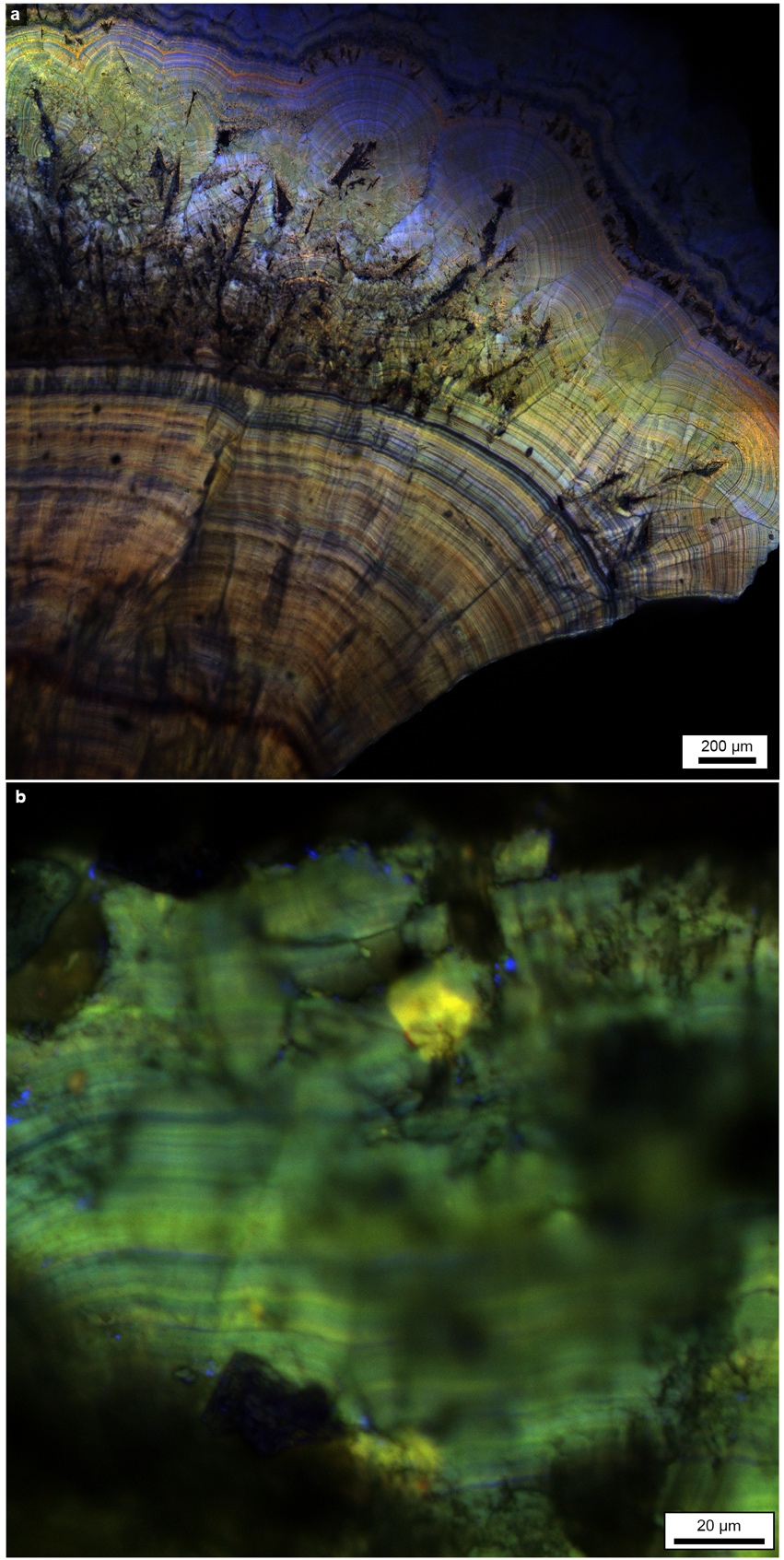


**Supplementary Figure 16 | Nano-layer AF is inherent to the MP-series kidney stones rather being than an artifact due to sample preparation. a**, CAF merged image of RGB emission channels showing COMC nano-layering in a random MP-series kidney stone polished with grit and embedded in epoxy. Epoxy vacuum impregnation was not performed (i.e., the epoxy did not penetrate into stone pore spaces). The stone surface was polished with 12, 6 and 3 µm grits using a Buehler EcoMet 300 grinding and polishing machine (Chicago, IL). Both epoxy and grit have AF characteristics. **b**, CAF merged image of RGB emission channels showing COMC nano-layering in a different MP-series kidney stone covered in JB-4 Plus, a non-AF polymer. In this preparation, no AF materials such as epoxy or grit were used. Yet, COMC dark and light AF nano-layering was visualized, indicating the original organic matter entrapped within each kidney stone cortex nano-layer has inherent RGB AF. Images (**a**) and (**b**) are displayed with best-fit intensity profiles.


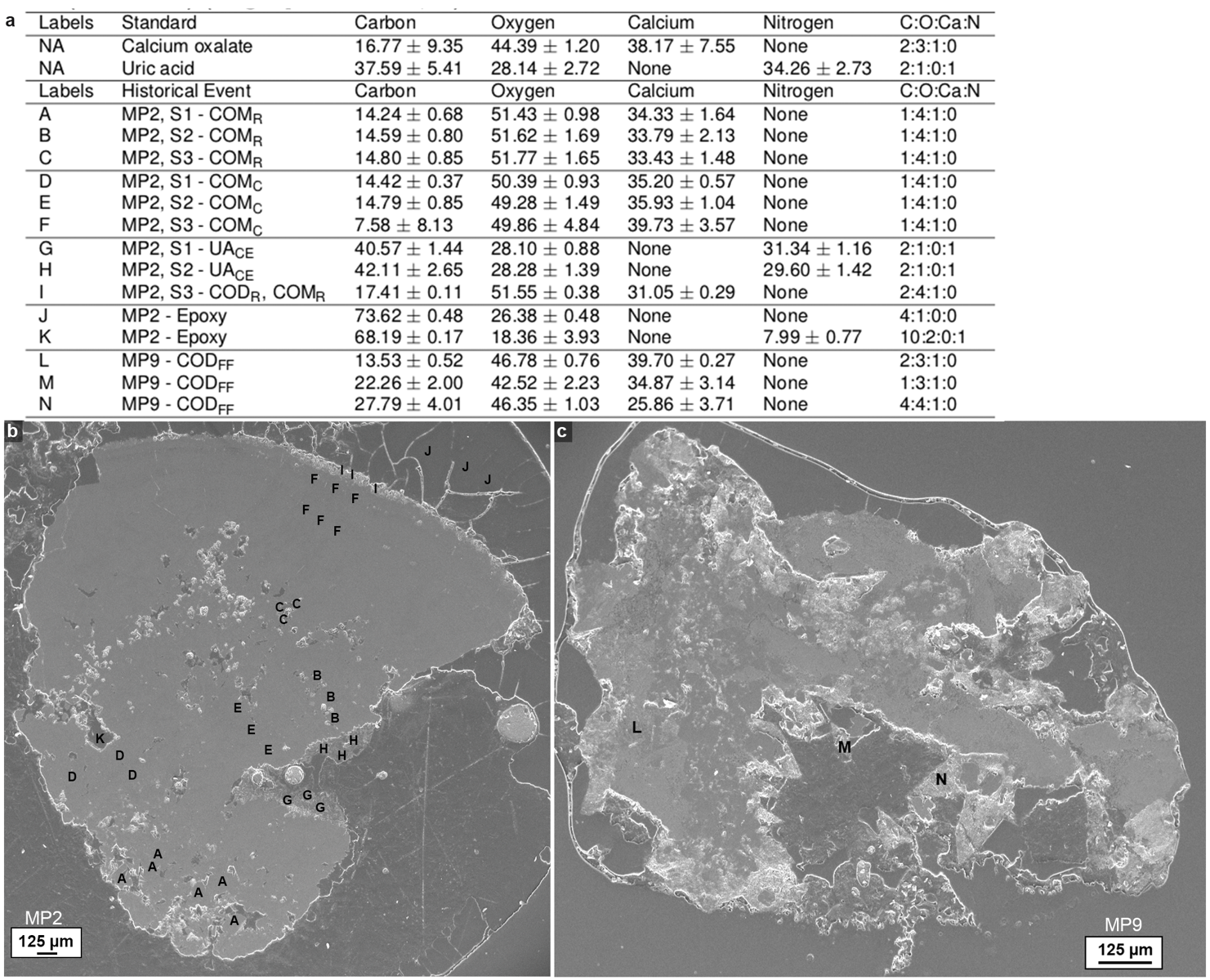


**Supplementary Figure 17 | Elemental composition of the Historical Sequence Events (HSE) within MP-series kidney stones. a**, Elemental composition of crystal fabrics at select locations indicated by A-N (weight percent + SD, %). **b**, Scanning electron microscope (SEM) image of MP2 stone (full optical image also shown in Supplementary Figure 1) showing locations of elemental analysis (A-K). **c**, SEM image of the MP9 stone (full optical image also shown in Supplementary Figures 9 p, q, r) showing precise locations of elemental analyses (L-N).


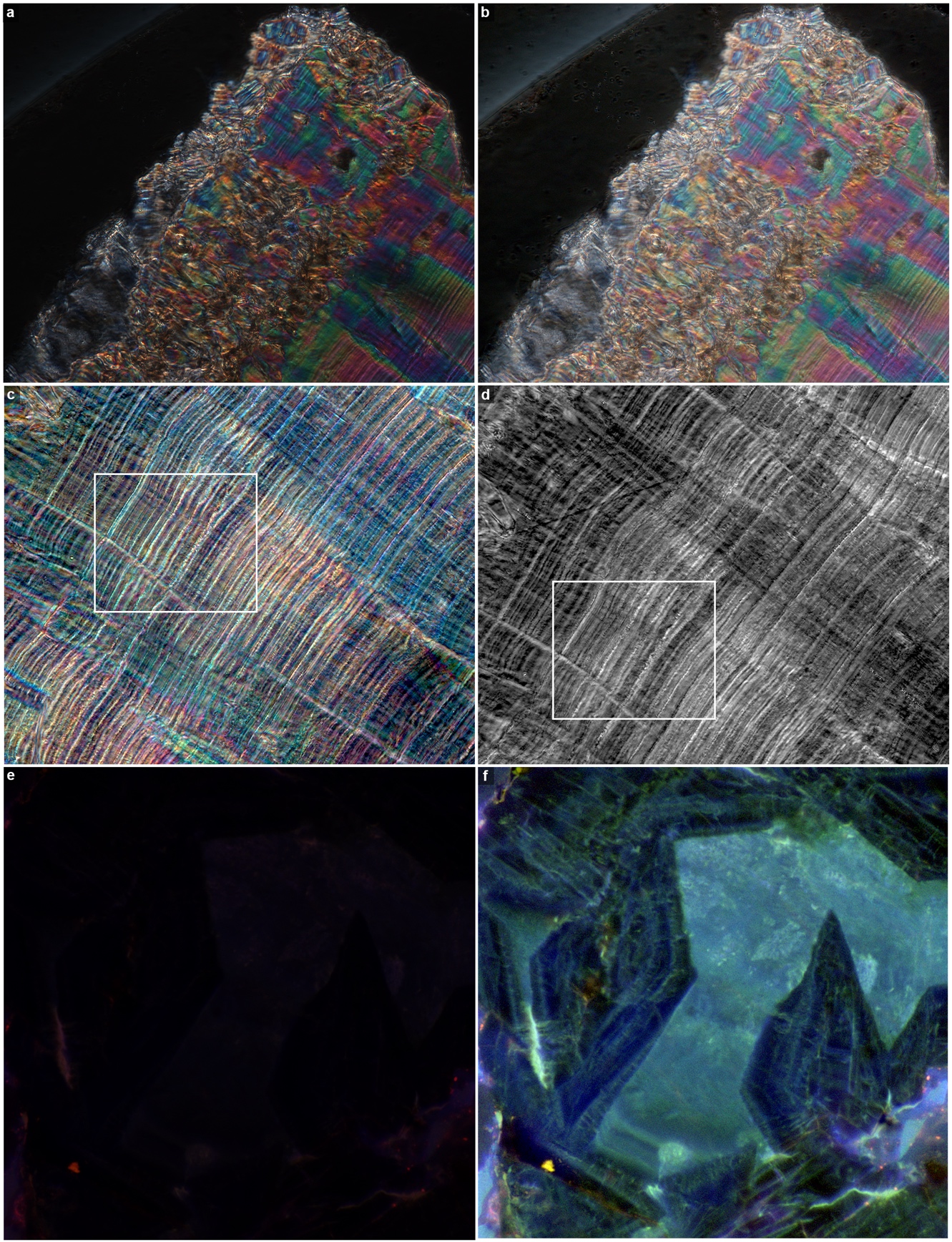


**Supplementary Figure 18 | Gamma adjustment scheme for polarization and some SRAF images of MP-series kidney stones. a,** Raw CPOL image. **b,** Image in (**a**) displayed with best-fit intensity profiles after a gamma correction of 0.45 in the Zeiss Zen program (Blue version). **c,** Color CPOLPC image displayed with best-fit intensity profiles after a gamma correction of 0.45. **d**, Similar location as in (**c**) is displayed as a black and white image. The location of the box in **d** is marked for comparison with Fig. 2f and the same box location in color is marked in (c). **e**, Raw SRAF image of merged pseudo-colored RGB channels. **f,** Image in (**e**) displayed with best-fit intensity profiles after a gamma correction of 0.45 in the Zen program (Blue version). The following images were adjusted as described in this figure: Supplementary Figures 3a, 4e and f, 5a-f, 8d and f, 10c, f, i, and l, and Supplementary Figure 12.


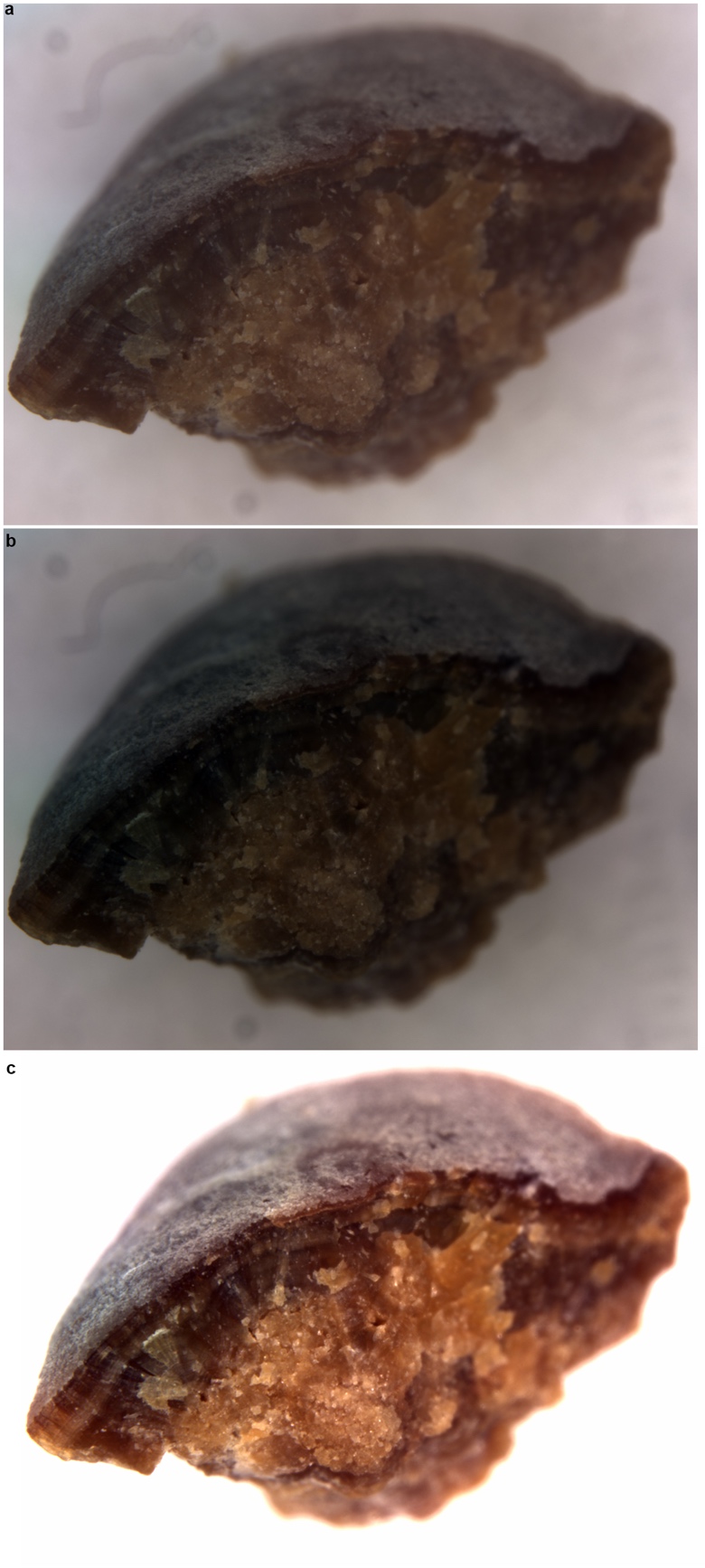


**Supplementary Figure 19 | Adjustments scheme for whole MP2 CaOx kidney stone bright field images. a**, Raw BF image. **b**, Image in (**a**) displayed with min/max intensity profiles. **c**, RGB channels of image in (**b**) adjusted together in the Zen program (Blue version) such that the maximum value is 7,591. The images in Supplementary Figure 9 were adjusted as described in this figure.


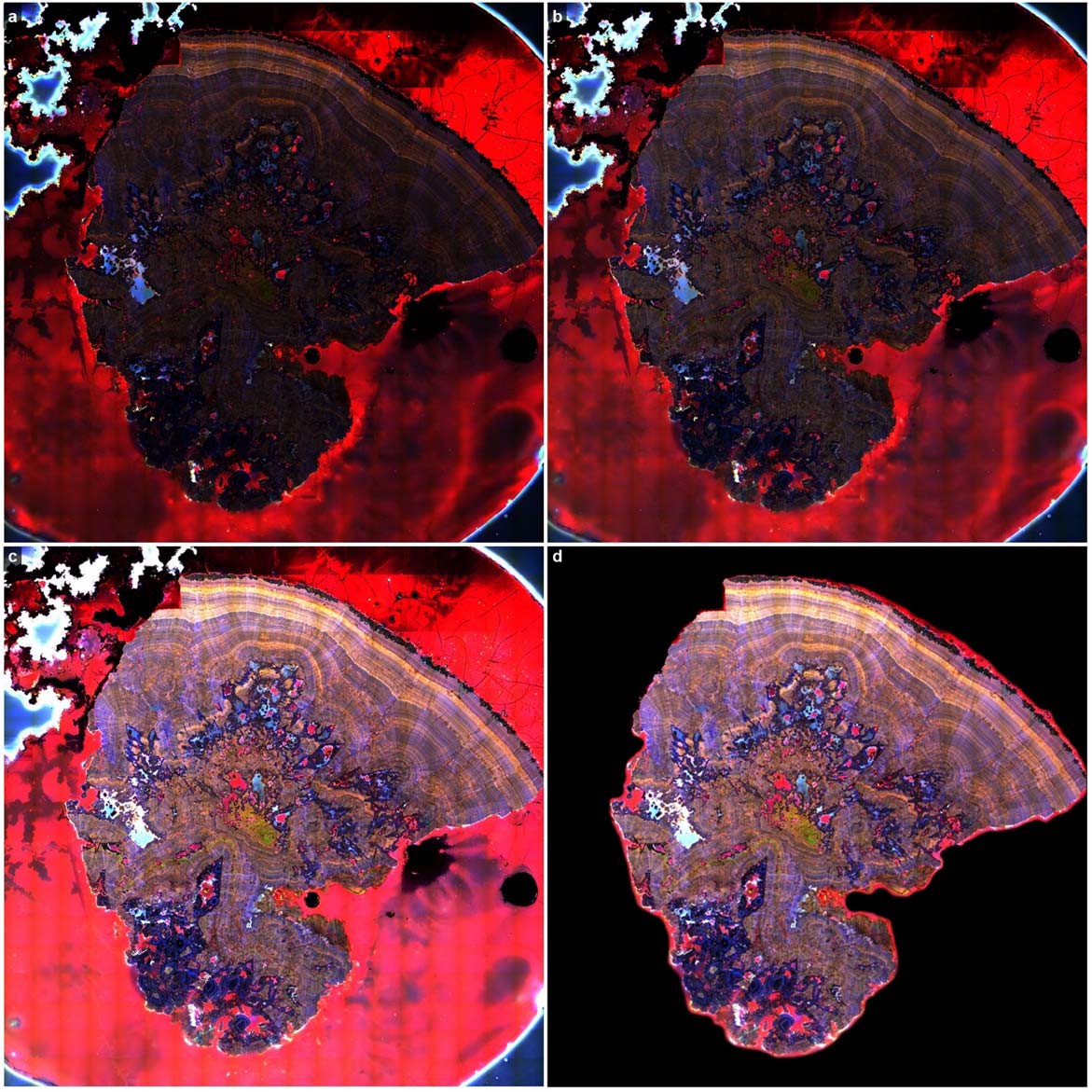


**Supplementary Figure 20 | Adjustments scheme for tiled whole MP2 CaOx kidney stone thin section. a,** Raw tiled CAF image of merged pseudo-colored RGB channels. **b,** Image in (**a**) displayed with best-fit intensity profile. **c,** Image in (**b**) with a gamma correction of 0.70 and maximum gray values of all three channels were adjusted together to 75. Adjustments in (**b**) and (**c**) are done in the Zen Program (Blue version). **d,** Image from (**c**) cropped and set to a black background together with smooth edges in the Adobe Photoshop program. The Supplementary Figure 1 was adjusted as described in this panel.


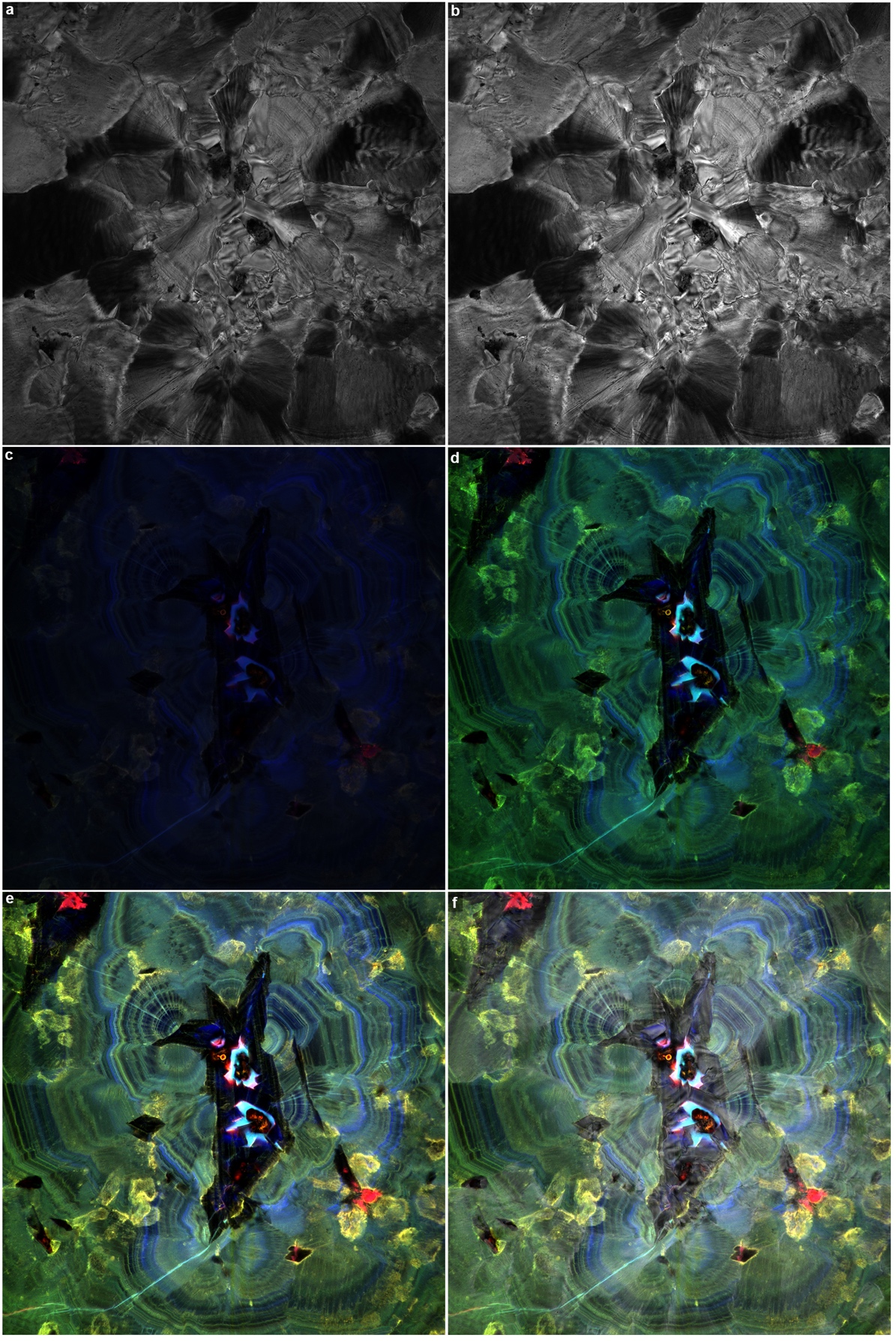


**Supplementary Figure 21 | Adjustments scheme for SRAF and BF image overlays of the MP2 CaOx kidney stone. a,** Raw BF image. **b,** Maximum gray value of (**a**) set to 150. **c,** Raw SRAF image of merged pseudo-colored RGB channels. **d,** Image from (**c**) displayed with best-fit intensity profile. **e,** Maximum gray values of blue, green and red channels from image (**d**) adjusted to 19,422, 6,877, and 6,710, respectively using the Zen program (16-bit gray images, Blue version). **f,** BF image (**b**) overlaid on the SRAF image (**e**) using ‘screen’ mode in Adobe Photoshop to highlight the COMR crystals filling inside the CODFF as indicated by the blue color used to depict HSE event 6 in Fig. 2b. Fig. 2c and the Supplementary Figures 2a-d were adjusted as described in this figure.


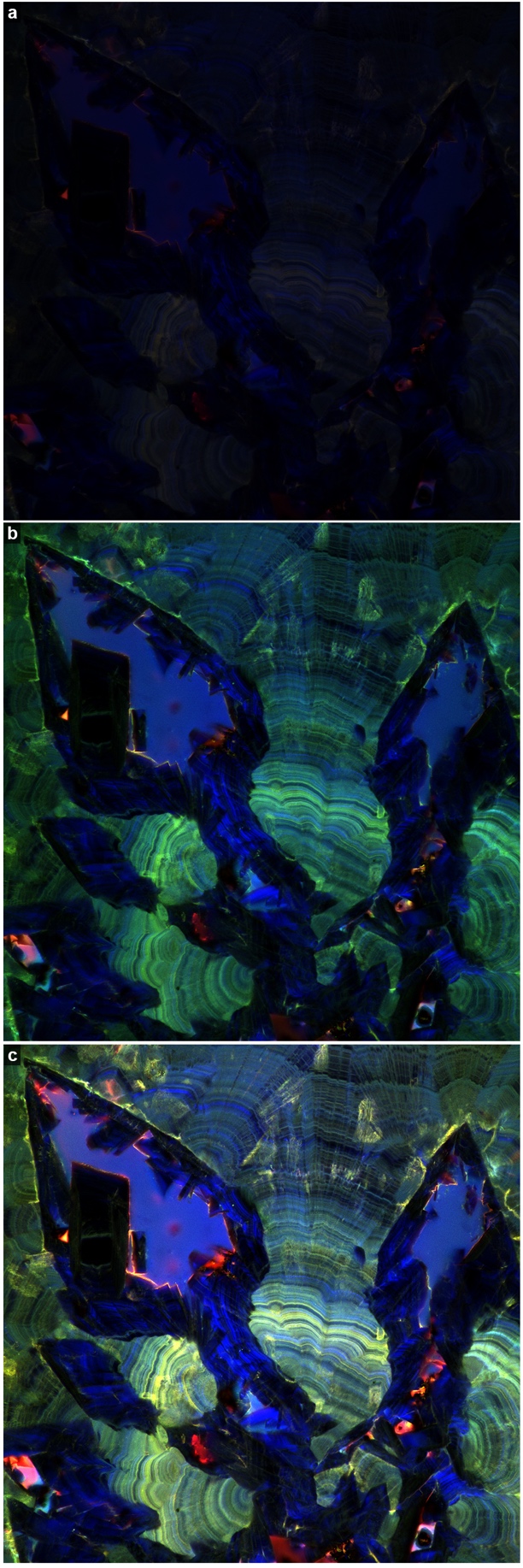


**Supplementary Figure 22 | Adjustments scheme for most SRAF images collected from the MP-series kidney stones. a,** Raw SRAF image of merged pseudo-colored RGB channels. **b**, Image from (**a**) displayed with min-max intensity profile. **c,** Maximum values of blue, green and red channels in image (**b**) adjusted to 14,780, 6,730, and 8,131, respectively in Zen program (Blue version). The following images were adjusted as described in this figure: Figs. 1c, 2b and c, Supplementary Figures 2g, 3b and c, 4a-d, 8b and c, 10a, c, d, f, g, i, j, l.

**IV. Supplementary Videos**

**Supplementary Video 1.** Animated version of tracing from Supplementary Figure 1 illustrating the MP2 CaOx stone is actually composed of an interlocked complex of three individual stone fragments. Tracing legend includes: dark blue = CODFF aggregates replaced by COMRcrystals; green = COMC; yellow = COMCE; gray = UACE; cyan = COMR and CODR.

**Supplementary Video 2.** SRAF optical sections of Fig. 2d showing ~140 nm nano-layering of COMC and radiating twinned crystals growing with their c-axis oriented perpendicular to each dark or light nano-layer.
